# Identification of new drug treatments to combat COVID19: A signature-based approach using iLINCS

**DOI:** 10.21203/rs.3.rs-25643/v1

**Published:** 2020-04-30

**Authors:** Sinead M O’Donovan, Hunter Eby, Nicholas D Henkel, Justin Creeden, Ali Imami, Sophie Asah, Xiaolu Zhang, Xiaojun Wu, Rawan Alnafisah, R. Travis Taylor, James Reigle, Alexander Thorman, Behrouz Shamsaei, Jarek Meller, Robert E McCullumsmith

**Affiliations:** University of Toledo; University of Toledo; University of Toledo; University of Toledo; University of Toledo; University of Toledo; University of Toledo; University of Toledo; University of Toledo; University of Toledo; University of Cincinnati College of Medicine; University of Cincinnati College of Medicine; University of Cincinnati College of Medicine; University of Cincinnati College of Medicine; University of Toledo College of Medicine and Life Sciences

**Keywords:** transcriptomic signatures, putative COVID-19 drugs, coronavirus-infected cell lines

## Abstract

The COVID-19 pandemic caused by the novel SARS-CoV-2 is more contagious than other coronaviruses and has higher rates of mortality than influenza. As no vaccine or drugs are currently approved to specifically treat COVID-19, identification of effective therapeutics is crucial to treat the afflicted and limit disease spread. We deployed a bioinformatics workflow to identify candidate drugs for the treatment of COVID-19. Using an “omics” repository, the Library of Integrated Network-Based Cellular Signatures (LINCS), we simultaneously probed transcriptomic signatures of putative COVID-19 drugs and signatures of coronavirus-infected cell lines to identify therapeutics with concordant signatures and discordant signatures, respectively. Our findings include three FDA approved drugs that have established antiviral activity, including protein kinase inhibitors, providing a promising new category of candidates for COVID-19 interventions.

## Introduction

Severe acute respiratory syndrome coronavirus 2 (SARS-CoV-2) is responsible for the first global pandemic in a decade, coronavirus disease 2019 (COVID-19)^1^. Initial reports of a novel SARS-like acute respiratory syndrome emerged in late 2019 from Wuhan, China^2^. Since then, COVID-19 has spread to over 150 countries and all continents except Antarctica3,4. At the time of writing, over one million people have been infected, over 60,000 deaths have been attributed to this outbreak4, and millions of additional infections are projected to occur globally in upcoming months^3^,^4^. COVID-19 is less infectious than SARS-CoV1 but more lethal than the common flu1 with an estimated mortality rate of 3.4%2. The incubation period, on average, is 5.2 days; in severe cases, the median time course from disease onset to death is 14 days^5^. While fever, cough, fatigue, and myalgias^6^–^10^ are common, mild presentations of COVID-19, the disease can fatally evolve into a severe pneumonia, complicated by acute respiratory distress syndrome, hypoxemic respiratory failure, and cytokine storm secondary to prolonged infection8. In addition to the significant medical burden imposed by this outbreak, it is estimated that the global economic cost of COVID-19 will be over $1 trillion in 2020^11^. The emotional toll on individuals will be incalculable, with prolonged quarantine policies restricting personal freedom and social contacts.

Current treatment is supportive and is focused on managing disease complications and secondary symptoms^12^–^14^. Drugs indicated for other infectious diseases, such as antiviral and antiparasitic therapies, have been used for COVID-19 patients, but there is a paucity of evidence supporting their efficacy^15^.

There is now an immediate need to identify therapeutic compounds that can be rapidly repurposed for COVID-19 treatment. Recent efforts to address this pressing public health concern include a comprehensive network-based approach to identify 16 candidate drugs (and drug combinations) that may be repurposed for the treatment of this disease^16^. To further expand this area of novel research, in the present study we employ a bioinformatics approach with the goal of datamining an extensive drug signature resource to distill a list of drug therapies that may prove fruitful in the search for COVID-19 therapies.

To accomplish this, we apply a signature-based connectivity analysis^17^–^19^ utilizing the extensive chemical perturbagen “omics” datasets deposited in the Library of Integrated Network-based Signatures (LINCS) database^17^,^20^,^21^. LINCS is a repository for systematically generated gene signatures based on the L1000 assay^22^. These gene signatures reflect cellular perturbations in response to pharmacological treatments; LINCS contains datasets for over 40,000 small molecules (drugs) in various cell lines. Different small molecules that produce signatures composed of highly similar patterns of gene expression changes, or “concordant” signatures, reflect shared connections between small molecules.

Here, we apply a two-pronged approach to identify novel compounds for the treatment of COVID-19. First, we identify pharmacologic therapies that are effective in the treatment of pathogens in the coronavirus family, like SARS and Middle East Respiratory Syndrome (MERS), as well as other viral illnesses^23^–^27^. We then generate gene signatures for these targets in iLINCS (http://ilincs.com) and highlight connected small molecule signatures to identify which of these candidate drugs are highly concordant with current therapies. Simultaneously, we generate gene signatures from coronavirus infected human cell line transcriptomic datasets. We analyze these data in iLINCS to directly match disease signatures with discordant small molecule signatures, thereby identifying drugs that “reverse” the disease signature. Finally, we compile a list of drugs from these two approaches to identify high-yield candidate drugs that may have therapeutic utility in the treatment of COVID-19.

## Results

**Figure 1:**
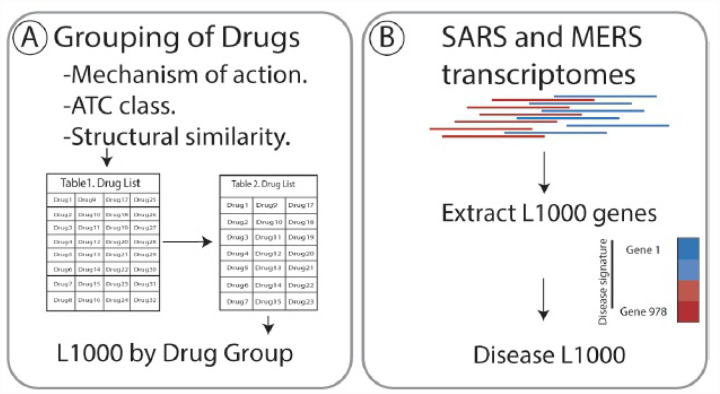

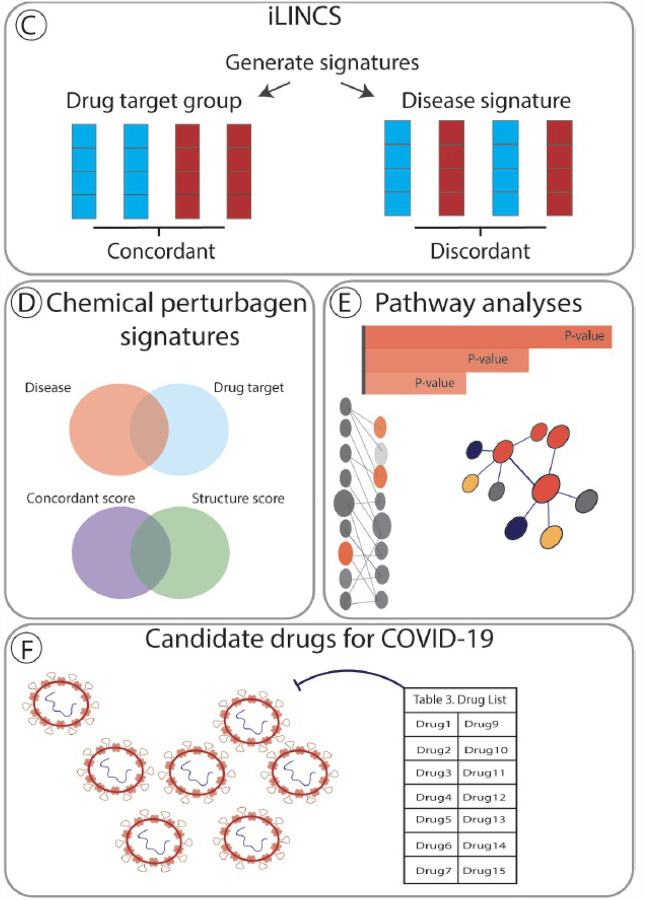
Overview of the workflow to identify candidate repurposable drugs to combat COVID-19. A) Drugs that are currently in use to treat coronavirus and putative COVID-19 treatments were clustered based on mechanism of action, ATC class and structural similarity. B) Gene expression data of the 978 genes that comprise the Library of Integrated Network-Based Cellular Signature (iLINCS) L1000 genes were extracted from severe acute respiratory syndrome (SARS) and Middle East respiratory syndrome- related coronavirus (MERS) (GSE56192) transcriptomic datasets. C) Consensus iLINCS gene signatures were generated for drug groupings and disease. D) Connectivity analysis was conducted and a list of chemical perturbagens that are concordant (≥ 0.321 concordance) to the drug target grouping signatures or discordant (≤ − 0.321 discordance) to the disease signatures was generated. Chemical perturbagens are curated to identify top candidate drugs. E) Biological pathways of drug target groupings, disease signatures and candidate drugs was conducted. F) Fourteen perturbagens with concordance scores ≥ 0.8 in MCF7 and HA1E cell lines were shortlisted as repurposable candidate drugs.

Applying the workflow outlined in Figure 1, we identified nine drugs, with known efficacy in treating coronavirus family pathogens, for which there are gene signatures in iLINCS (Table 1 and extended information in Table S1). These drugs were clustered into five groupings according to their mechanism of action, Anatomical Therapeutic Chemical (ATC) classification and/or structural similarity (Table 1).

**Figure 2:**
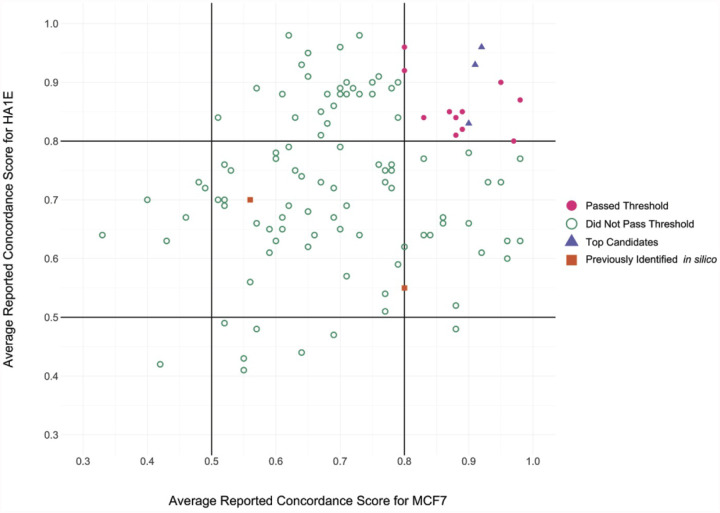
Scatter Plot of average reported concordance scores for candidate drugs in the MCF7 cell line and HA1E cell line. A total of 112 drugs with concordance scores > 0.321 were common between both cell lines (open circles). Fourteen drugs were identified with concordance scores ≥ 0.8 in both cell lines (closed circles). Drugs above this threshold are considered “candidate” drugs. Top candidate drugs, those approved for use in humans and with demonstrated antiviral activity in vitro, are also identified (triangles). Drugs identified by other in silico drug screening studies that were also found in our analysis are identified (squares).

Simultaneously, we extracted differential gene expression data on the 978 genes that comprise the L1000 from a publically available SARS (GSE56192) transcriptomic dataset. Gene signatures composed of genes changed LFC ≥ 0.5 and ≤ −0.5 were generated for the disease signature (Table S2). In iLINCS, we conducted connectivity analysis to identify chemical perturbagens that are highly concordant to the drug target groupings (≥ 0.321) or highly discordant to the disease signatures (≤ −0.321), established minimum iLINCS concordance score cutoffs 22,28. This resulted in identification of 112 chemical perturbagens common to two cell lines, MCF7 and HA1E (Figure 2). Fourteen chemical perturbagens were identified at concordance scores ≥ 0.8 in both cell lines and were considered “candidate” drugs for the treatment of COVID-19 (Table 2). The Tanimoto scores for the candidate drugs and the original 9 drug targets were generated, showing structural similarity between drugs currently in use for the treatment of coronavirus family pathogens and our newly identified candidate drugs (Figure S1).

Table 2 Candidate drugs are separated into two cohorts: drugs with reported antiviral activity and those with no reported antiviral activity. These drugs are then subcategorized as clinically relevant (used in human subjects) or experimental (used in research but not yet approved for humans). Concordance scores in the MCF7 and HA1E cell lines represent the average concordance scores between the identified candidate drug and at least 2 of the drug target clusters in that cell line. All 14 candidate drugs have an average concordance ≥ 0.80 in both cell lines. DrugBank I.D. and Mechanism of Action (MOA) is referenced from Drug Bank (https://www.drugbank.ca/). Drug Class is referenced from the second level of the Anatomical Therapeutic Chemical (ATC) classification (https://www.whocc.no/atc_ddd_index/). For “Experimental drugs”, the MOA was cited from iLINCS, or alternatively, the perturbagens top two significant Gene Ontology (GO) Molecular Functions are listed under MOA and denoted with the superscript “#”. “^” Indicated and approved for use only by the European Medicines Agency. HSV, Herpes Simplex Virus; HIV, Human Immunodeficiency Virus; LV, Lassa Virus; ASFV, African Swine Fever Virus; YF, Yellow Fever; DF, Dengue Fever; CV, Chikungunya virus; SARS, Severe Acute Respiratory Syndrome virus; MERS, Middle East Respiratory Syndrome virus.

Unsupervised clustering of L1000 disease gene signatures demonstrates significant differences in patterns of gene expression induced by SARS, MERS and influenza (Figure S2). Influenza is utilized as a control dataset as it represents a non-coronavirus pathogen that also causes respiratory illness. As expected, unsupervised clustering of L1000 gene signatures shows discordance between disease signatures and drug target grouping signatures, which are comprised of drugs utilized to treat SARS and to a lesser extent, MERS (Figure S3–4).

Biological pathways analysis demonstrated a range of perturbations, including those in similar biological pathways (immune system pathways and cell cycle processes) induced by both drug target groupings and disease signatures (Figure S5–6). Unsupervised clustering of L1000 gene signatures also shows discordance between SARS (and MERS) disease signatures and the identified candidate drug signatures (Figure S7–10), including those with antiviral properties.

Biological pathway analysis indicates that the identified candidate drugs also induce changes including in similar biological pathways as disease signatures (immune system and cell cycle related pathways) (Figure S11–12).

Thus, we distilled a list of drugs, derived from pharmacological and disease perturbation signatures that may have therapeutic utility in the treatment of COVID-19. The candidate drugs identified are:

### Tyrosine Kinase Inhibitors.

Tyrosine kinases are essential for viral RNA synthesis, viral ribonucleoprotein nuclear export, and virion release. Inhibitors that target this protein class may, therefore, demonstrate activity against viruses^49^.

Genistein is an isoflavonoid derived from soy-products that has been implicated as an antiparasitic^50^ and antineoplastic agent51 in humans. Several clinical trials of Genistein are ongoing to treat prostate, breast, and bladder cancers52,53. Genistein also has potent antiviral activity in a number of *in vitro* models. It has efficacy against RNA viruses from different families, such as filoviridae (Ebola virus), feoviridae (rotavirus), and arenaviridae (Lassavirus, Pichindé virus)^34^,^35^,^38^,^39^; retroviruses like HIV-1^36^,^37^ and DNA viruses. Both *in vivo* and *in vitro* studies of Genistein demonstrated activity against African swine fever viruses^40^ and Epstein- Barr virus^54^. While the pre-clinical evidence is promising, Genistein has yet to be explored as an antiviral therapy in humans.

AT9283 is a broad protein kinase inhibitor^55^,^56^. Canonically, it acts as a receptor and nonreceptor tyrosine kinase inhibitor but also effectively inhibits serine/threonine kinases, Aurora A/B kinases, Janus kinases (JAK) 2/3, and ABL kinases^57^. In a clinical setting, AT9283 has been predominantly studied as an antineoplastic for hematologic malignances and several trials are underway^58^–^60^. AT9283 has not been explored directly as an antiviral, although the ABL kinase inhibitor, Imatinib, is efficacious in preventing coronavirus (SARS-CoV and MERS-CoV) viral fusion with endosomes, effectively halting viral activity^61^. Given its role as a broad-spectrum kinase inhibitor, researching the antiviral properties of AT9283 may prove fruitful.

### Serine Threonine Kinase Inhibitors.

Alvocidib (also known as flavopiridol) is a pan-specific cyclin-dependent kinases (CDK) inhibitor that inhibits CDK1, CDK2, CDK4, CDK5, CDK6, CDK7, and CDK9^62^. Alvocidib is under clinical investigation as an antineoplastic for both solid tumors and hematologic malignancies^63^. Like other CDK kinase inhibitors, Alvocidib has been implicated as a broad antiviral against several DNA virus families^64^. Alvocidib has been studied as an inhibitor of transcriptional activation and elongation in the infectious lifecycle of DNA viruses (HSV-1, HSV-2) and retroviruses (HIV)^29^–^32^ and also suppresses replication of Influenza A^33^. This suggests that Alvocidib is a strong candidate drug for repurposing.

The antiviral activity of the following serine/threonine kinase candidate drugs have yet to be studied directly and further research is required to determine their potential as repurposable antiviral therapies. However, these candidates are members of drug families with demonstrated antiviral or antimicrobial properties that could be exploited for the treatment of COVID-19.

GSK-1059615 is a reversible, ATP-competitive, thiazolidinedione inhibitor of phosphoinositide 3-kinase (PI3K) and has been studied as an antineoplastic for solid tumors^65^. Though the antiviral activity of GSK-1059615 has yet to be determined, the thiazolidinedione drug family has a broad range of antibacterial and antiparasitic activity^66^–^68^. Idelalisib is a phosphoinositol 3-kinase (PI3K)/protein kinase B (AKT) signaling inhibitor^69^–^71^. *In vitro* experiments show that the downstream target pathways of kinase inhibitors like Idelalisib, extracellular signal-regulated kinase (ERK)/mitogen-activated protein kinase (MAPK) and PI3K/AKT/mammalian target of rapamycin (mTOR) signaling responses, are specifically modulated during infection with coronavirus pathogen MERS^72^. Thus, inhibiting this virulent signaling pathway using kinase inhibitors is potentially an efficacious therapeutic strategy.

### Antioxidants and Antimicrobials.

Ivermectin is a promising drug candidate for COVID-19. It is a well-characterized anthelmintic for *Onchocerca volvulus*, the causative parasitic roundworm of “river blindness” or the “black sight”^73^–^75^. Canonically, Ivermectin works as a chloride-channel agonist^76^. Ivermectin has a well-established safety profile in humans and has been under investigation for repurposing in various parasitic diseases, cancers, neurological disorders, and viral infections^75^–^77^. The efficacy of Ivermectin in the treatment of RNA virus families, such as flaviviridae^41^,^43^,^78^,^79^ and togaviridae^43^ has been demonstrated *in vitro*. Of note, Ivermectin has been used as an adjunct therapy in patients with HIV and concomitant parasitic infections^80^,^81^.

*In vitro*, Ivermectin has shown efficacy in targeting HIV^41^ alone; by inhibiting HIV-1 integrase Ivermectin potentially prevents the viral genetic material from entering the host genome^42^. Efficacy for Ivermectin as an antiviral in humans warrants further investigation, especially as the global COVID-19 pandemic ensues.

Idebenone is a synthetic derivative of ubiquinone, also known as Coenzyme Q10^82^,^83^. This drug acts to increase the production of ATP by enhancing oxidative phosphorylation. As a general antioxidant, Idebenone may prevent lipid peroxidation, reduce membrane oxidative stress, and scavenge free radicals^84^. Idebenone has been used for a number of human neurodegenerative disorders, and its safety has been validated^85^,^86^. Antioxidants such as Idebenone have been hypothesized to mitigate the deleterious effects of a “reactive-oxygen species burst”^45^ from viruses with a pulmonary and respiratory predilections (Influenza and SARS)^44^. However, further work is required to determine the utility of antioxidants like Idebenone as adjunct treatments for COVID-19.

Penicillin V is a beta-lactam antibiotic and is indicated primarily for treating gram-positive bacterial infections like Treponema pallidum, the causative organism of syphilis^87^. Penicillin binds to a family of bacterial transpeptidases, termed penicillin binding proteins, which effectively inhibit cross-linking of peptidoglycan in the bacterial cell wall^87^. To the best of our knowledge, penicillin V does not have an experimental or clinical indication in the treatment of viruses. In addition, antibiotics such as penicillin should be employed judiciously, given their well-characterized ability to induce hypersensitivity reactions^88^,^89^. However, like azithromycin, utilizing penicillin as an adjunct therapy may be advantageous to empirically cover bacterial infections co-morbid with viral infections.

### Candidate Drugs with Unknown Utility.

Finally, five drugs (AC1MJ3VH, Broad-Sai-595, CHEMBL2136735, COT-10B and GSK Inhibitor IX) with limited or no known biological function were identified. These drugs do not have identifiers in Drug Bank. They were not considered to be of utility as candidate drugs to repurpose for treatment of COVID-19.

## Discussion

The COVID-19 outbreak is an escalating public health concern that requires a swift and comprehensive response. Research is progressing rapidly. There are currently over 200 clinical trials exploring a range of pharmacological and non-pharmacological options for the treatment of COVID-19, but as of yet, there are no vaccines or therapies approved specifically for this disease. In recent months, a number of *in silico* studies addressing this gap in our knowledge have identified putative repurposable drugs for treating COVID-1916,^90^–^93^. Many of these studies exploit the recent finding that SARS-CoV-2 may enter the cell by binding to angiotensin converting enzyme 2 (ACE2)^94^ and utilize a combination of structural and biomedical data to identify drug candidates. One such study used artificial intelligence algorithms (BenevolentAI) to identify the JAK inhibitor Baricitinib^90^. Baricitinib may reduce the ability of the virus to enter cells and is currently in clinical use as a treatment for rheumatoid arthritis. To advance therapeutic discovery and the identification of candidate drugs for COVID-19, we employ an alternative, signature-based bioinformatic approach. In this study, we data mine the extensive LINCS database, which acts as a repository of “L1000” gene signatures generated by treating various cell lines with over 20,000 small molecules. The L1000 genes are a reduced representation of the transcriptome, a method by which a select group of genes account for ~82% of the information content of the transcriptome^95^. The approach involved feature selection/reduction techniques applied to 12,063 gene expression samples profiled on microarrays from GEO^96^. Benchmarking of the L1000 assay versus RNAseq yielded a cross-platform correlation of 0.8495, suggesting the L1000 assay represents an efficient alternative to RNA-Seq.

Utilizing this resource, our two-pronged connectivity analysis approach identified candidate drugs that are 1) highly concordant to current drugs employed to treat coronavirus family pathogens and 2) highly discordant to SARS disease gene signatures. As there are currently no publically available datasets for human (or other) tissues infected with SARS-CoV-2 virus, we generated a disease signature from an RNAseq dataset of human lung cells infected with SARS; SARS and SARS-CoV-2 are highly homologous, sharing envelope and nucleocapsid protein sequence identities of 96% and 89.6%, respectively16.

The main class of drugs identified from our analyses are kinase inhibitors. Kinase inhibitors are high-yield targets, with new small molecule kinase inhibitors being developed every year and over two dozen small molecule kinase inhibitors already approved for human use^97^. Their potential as antiviral treatments has also been explored in recent years^91^,^98^–^100^. Viruses depend on host cell protein kinases for every step of their life cycle, including viral entry into the cell, cell cycle processes and cellular stress response^101^. Thus, targeting these protein kinases using kinase inhibitors will disrupt the virus’s ability to hijack cellular processes. As many host protein kinases are broadly required by different viruses, kinase inhibitors are excellent candidates for broad-spectrum antiviral therapies^99^. Our study identified two kinase inhibitors, Genistein and Alvocidib. These drugs have demonstrated, extensive, antiviral properties *in vitro*^30^,^40^ and both are approved for use in humans, making them strong candidate therapies for the treatment of COVID-19.

Kinase inhibitors AT9283, GSK1059615 and Idelalisib were identified by our analyses and are approved for use in humans, but their antiviral properties have yet to be tested directly. These drugs inhibit a range of different protein kinases including PI3K/mTOR, GSK3β and ABL kinases. *In vitro* work demonstrates that inhibitors targeting these kinases are highly effective at treating coronavirus pathogens SARS and/or MERS^61^,^72^,^102^,^103^, in addition to other viral pathogens^104^–^108^. However, further work is required to determine the specific antiviral profiles of these candidate drugs, before they can be considered for repurposing.

Interestingly, we, and another group, identified Ruxolitinib, a JAK kinase inhibitor that is utilized as an antineoplastic^91^. Although Ruxolitinib was not identified above the same stringent threshold as our top candidate drugs, its discovery by two different *in silico* approaches suggests that this kinase inhibitor may have utility as a candidate drug for treating COVID-19. Importantly, kinase inhibitor Barcitinib is now undergoing clinical trial in response to the COVID-19 pandemic (NCT04320277). Thus, kinase inhibitors represent an expanding, if underexplored, avenue of research for the treatment of viral illnesses, including coronaviruses. Repurposing kinase inhibitors, many of which are already approved for use in humans, is a time-and cost-effective method to identify new therapeutics in a rapidly evolving situation such as the one posed by the current outbreak of COVID-19.

Another strong candidate drug finding from our analyses is the antimicrobial Ivermectin. Best known for its efficacy in treating “river blindness,” Ivermectin is already in widespread use as an anthelmintic and was recently shown to have potent antiviral characteristics *in vitro*, in experiments targeting flaviviruses and RNA viruses^76^. Indeed, Ivermectin is used as an adjuvant therapy in patients with HIV and a concomitant parasitic infections^80^,^81^. In phase II/III clinical trial, Ivermectin safely and significantly reduced Dengue viral NS1 protein serum levels 109,110.

Although no clinical benefit was seen, the dosing regimen may yet be optimized based on pharmacokinetic and pharmacodynamic data^109^. In addition, Ivermectin shows potent antiviral activity for SARS-CoV-2, reducing viral RNA approximately 5000-fold in infected cells at 48 hours, in a recent *in vitro* study^111^. Taken together, Ivermectin’s established safety profile and efficacy in reducing SARS-CoV-2 viral material *in vitro* suggest this drug is worthy of further consideration as a treatment for COVID-19.

A number of our other candidate drug findings may also prove useful as adjuvant treatments for the secondary effects associated with viral infection, including the antioxidant Idebenone, and the antibiotic Penicillin^112^. During the 2009 H1N1 influenza pandemic, bacterial infection was a suspected, underreported contributor to patient hospitalization and death^113^.

Interestingly, the antibiotic quinacrine was also identified in a recent network-based COVID-19 drug screen^16^ and another β-lactam antibody ceftriaxone, is in clinical trial for (adjunct) treatment of COVID-19 (NCT02735707). The immunosuppressant Sirolimus was identified in our study, albeit at a less stringent threshold, as well as in another *in silico* drug screen^16^, as a candidate repurposable drug for treating COVID19. Immunosuppressants may address the symptoms resulting from overactivation of the immune system (“cytokine storm”) in response to COVID-19 infection^114^, and this class of drug is also in clinical trial as adjunct treatments (e.g. Thalidomide NCT04273529).

### Limitations

The antimicrobial drugs that comprise our drug target groupings are limited to those that have gene signatures in iLINCS. We analyze gene signatures generated in two cell lines, MCF7 and HA1E, as data was available for all of our drug targets in these cell lines only. Our drug target clusters may induce different patterns of gene expression changes in other cell lines, resulting in different gene signatures, and potentially, the identification of different chemical perturbagens. We utilize a single, representative SARS transcriptomic disease signature to identify discordant chemical perturbagens, as no transcriptomic SARS-CoV-2 datasets are available at the time of writing. Although the two viral genomes are highly similar (envelope and nucleocapsid protein share sequence identities of 96% and 89.6%, respectively), we cannot exclude that chemical perturbagens that are discordant to SARS may not have the same level of discordance to SARS-CoV-2. Finally, as with other *in silico* screening approaches, the candidate drugs identified here are not necessarily ready for human use. Several of the candidate drugs are used in the treatment of human disease, but not viral infections or COVID-19 specifically, and require further investigation for dosage, efficacy etc. A subset of the drugs were explored in experimental, but not clinical settings, and are not currently approved for use in humans.

In summary, our approach has identified candidate repurposable drugs, from the >20,000 small molecules in the LINCS repository, that may be utilized to combat COVID-19. Several of the candidate drugs are 1) currently approved for use in humans, 2) have demonstrated antiviral efficacy *in vitro* and 3) a number were also identified in other *in silico* analyses. Our findings contribute to the relatively novel literature addressing the purported broad-spectrum antiviral efficacy of kinase inhibitors and may offer a novel avenue for investigation in the search for COVID-19 therapies. While there is evolving evidence for kinase inhibitors as antivirals, other antimicrobials could be repurposed as well. Finally, our bioinformatic workflow identified an antioxidant and two known antimicrobial drugs, which are concordant with current therapies being explored to combat SARS-CoV-2.

## Methods

### Selecting and grouping antimicrobials with known efficacy in treating coronavirus family pathogens

The workflow for this study is outlined in Figure 1. We conducted a PubMed search using search terms “coronavirus” or “COVID-19” and “antiviral” or “drug” or “therapy” and generated a list of compounds utilized to treat coronavirus family pathogens or identified as putative COVID-19 therapeutics. We identified seventeen drugs for potential analysis (Table S1). L1000 gene signature datasets were available for nine of the seventeen drugs (Table 1) using the integrative web platform iLINCS (http://ilincs.com). The iLINCS L1000 hub gene assay assesses genome-wide transcriptional changes following perturbation by one of more than 20,000 small molecules95. Eight drugs without signatures were excluded from further analysis. Gene signatures were generated for all remaining drugs. To standardize our analysis, we used the two cell lines that appeared most frequently in the signatures: MCF7 and HA1E. Hydroxychloroquine has an MCF7 signature only. For further standardization, where possible, signatures for a 24-hour time point and 10 uM concentration conditions were used.

Next, we grouped the nine drug targets based on canonical mechanism of action, the Anatomical Therapeutic Chemical (ATC) classification, and structural similarity. Drugs were grouped together if they were categorized by at least two of the three methods. The database DrugBank (https://www.drugbank.ca/) was used to group the drugs by their canonical mechanisms of actions.

Drug identification was only referenced from Drug Bank I.D. If no Drug Bank I.D. was available, this is indicated in Table 1 and Table S1. If there was no listed MOA from Drug Bank, then the MOA was appropriately cited, referenced from iLINCS, or was referenced from Gene Ontology (GO) Molecular Function 2018 accessed via Enrichr (http://amp.pharm.mssm.edu/Enrichr/enrich). Next, drugs were classified based on the ATC classification system (https://www.whocc.no/atc_ddd_index/). If a particular drug did not have an ATC classification, it was marked as “unclassified.” From DrugBank, we also collected the clinical indications, gene targets, and trade names. In addition, we probed the ATC Index (https://www.whocc.no/atc_ddd_index/) to identify the first- and second-level of drug classifications. The first-level classification was used to confirm drug grouping. Finally, to group drugs based on structural similarity, the structural data files (sdf) for the nine drugs under investigation were downloaded from DrugBank. The package ChemmineR was used to generate 1024-bit binary fingerprints for each compound. The Tanimoto coefficient between all pairs of fingerprints were then computed also using the R package “ChemmineR”. The Tanimoto coefficient, also known as the Jaccard similarity, represents the most popular measure for chemical similarity^115^ and is the ratio of the intersection of the two fingerprints divided by the union of the two fingerprints. The data were visualized using the “Corrplot” package. With a final list of drug clusters, the individual drug signatures within each grouping were collected and averaged across the L1000.

### Generating iLINCS gene signatures

Using the iLINCS portal, we acquired the LINCS chemical perturbagen signatures (978 genes that comprise the L1000) for each drug candidate. Genes with a log fold change (LFC) value of ≥ 0.85 or ≤ −0.85, indicating differential gene expression induced by the drug target compared to a corresponding control cell line, were exported to Microsoft Excel. Gene lists were pooled and averaged such that a master list of differentially expressed genes was generated for each drug candidate family. For example, genes with a LFC ≥ 0.85 or ≤ −0.85 that appeared in both the hydroxychloroquine gene signature and the chloroquine gene signature were averaged to calculate mean values for each differentially expressed gene in drug target grouping 1.

Next, the upregulated genes (LFC ≥ 0.85) were clustered and the downregulated genes (≤ −0.85) were clustered. These clusters were uploaded as user generated signatures into iLINCS. Next, we identified connected chemical perturbagens, utilizing a concordance threshold score of ≥ 0.321, an established minimum concordance score cutoff 22,28, to identify chemical perturbagen signatures that are considered highly correlated with our drug target grouping signatures.

### Gene signatures coronavirus-family induced disease datasets

At the time of writing, there are no publically availably Severe Acute Respiratory Syndrome Coronavirus 2 (SARS-CoV-2; COVID-19) transcriptomic datasets available. Thus, SARS (GSE56192) and Middle East Respiratory Syndrome (MERS; GSE56192) RNAseq datasets were identified in the Gene Expression Omnibus (GEO: https://www.ncbi.nlm.nih.gov/geo/). SARS and MERS are coronavirus family members that cause severe respiratory illnesses^116^. We selected these datasets based on the viral type, transcriptomic platform, and infected tissue type. The SARS genome in particular is highly homologous to SARS-CoV-2, the virus responsible for COVID-19^117^. The envelope and nucleocapsid proteins of SARS-CoV-2 are two evolutionarily conserved regions, with sequence identities of 96% and 89.6%, respectively, to SARS^16^. The selected datasets focused on evaluating transcriptomic changes post-infection in lung cell lines, the primary tissue affected by the viral infection in humans. Each selected dataset represents a single time point from each study. We selected the most representative time points based on the top 50 differentially expressed genes from each dataset. The time point selected for these datasets is 24h.

We conducted differential gene expression analysis of these datasets comparing virus infected samples to corresponding control samples. RNAseq raw count data were analyzed using edgeR R package v.3.28.1 for differential gene expression^118^. As a quality control step, we require that a gene have a count of at least 10 in at least some libraries before it is considered to be expressed. Normalization was performed using the default method, trimmed mean of M-values (TMM). This step is performed by using the calcNormFactor function, which returns the DGEList argument with a set of calculated normalization factors, one for each sample, to eliminate composition biases between libraries. org.Hs.eg.db R package v.3.10.0 was used to complement the gene annotation. Following analysis of the disease transcriptomic datasets, the subset of genes that comprise the LINCS L1000 were extracted from the differentially expressed gene list for each dataset. When the L1000 genes were extracted from the SARS and MERS RNAseq datasets, 944 and 947 overlapping genes, respectively, were extracted. We used the existing gene raw counts from GEO and did not remap the genes. Thus, if some of the L1000 genes were not mapped we were unable to access their expression. In addition, we applied a gene raw count cutoff of 10 as a quality control step, which also reduced the number of L1000 genes found in the final differential expression gene lists.

The extracted L1000 genes were uploaded into iLINCS. Genes with LFC in expression within three thresholds, 0.26 LFC, 0.5 LFC and all L1000 genes, were identified with a custom R script for further processing. As described above, upregulated and down regulated disease gene signatures were generated for each disease dataset (within each threshold) and uploaded into iLINCS to identify connected perturbagens. For disease gene signatures, chemical perturbagen signatures that are highly discordant (discordance score ≤ −0.321), indicating these perturbagens may “reverse” the disease signature, were identified. Genes at LFC ≥ 0.5 and ≤ − 0.5 threshold were then carried forward for further analysis. Utilizing this gene threshold generated optimal SARS disease signatures to identify a large number of discordant chemical perturbagens. In addition, we identified a microarray dataset of human airway epithelium cells infected with influenza A of the H1N1 serotype (GSE47963, 36h time point). The microarray data were extracted using GEOquery R package v.2.54.1 119. Specifically, SOFT format files from GEO containing all of the information in the GEO records were parsed. The normalized gene expression values associated with SOFT files for selected samples filewere then analyzed using limma R package v.3.42.2, linear model for microarray^120^. As described above, the L1000 genes were extracted. Since this dataset was performed on the microarray platform, which limits the gene detection to the probes available in the arrays, we extracted 968 L1000 genes.

In addition to the MERS dataset, which represents a pathogenic coronavirus like SARS, the influenza dataset was used as a comparison dataset in unsupervised clustering and biological pathway analyses (described below). Influenza A represents a non-corona family virus that also causes respiratory disease^121^. A total of 906 overlapping L1000 genes were used in clustering analysis and heat map generation.

### Identification of candidate chemical perturbagens (drugs) to treat COVID-19.

Candidate drugs were identified from the chemical perturbagen connectivity analysis using a custom script in R. The script downloaded the data from the iLINCS API and used the following criteria: Chemical perturbagens had a concordance score ≥ 0.321 compared to drug target grouping signatures or a discordance score ≤ −0.321 compared to disease signatures in cell lines MCF7 (6373/14355) or HA1E (4179/8141). If the same chemical perturbagen is identified multiple times, from different experimental conditions, replicate findings are removed so that only the highest concordance score (or lowest discordance score) for each chemical perturbagen remains. Chemical perturbagens were identified in the SARS disease signature analysis AND at least 2/5 drug target grouping signature analyses. This resulted in 506 chemical perturbagens from cell line MCF7 and 411 chemical perturbagens from cell line HA1E.

In total, 112 chemical perturbagens were common to both cell lines. Chemical perturbagens with concordance scores ≥ 0.8 in the MCF7 AND HA1E cell lines are considered “candidate drugs,” resulting in a final list of 14 candidate drugs. Heat maps of the L1000 gene data for each of the drug target groupings, candidate drugs and disease (SARS and MERS) datasets were generated as described above. In addition, the L1000 gene data for Furosemide (FUR), a drug which is not used to treat viral pathogens or related illnesses, and influenza, a non-coronavirus family pathogen associated with respiratory illness, were included in the heat maps. These datasets acts as controls, suggesting that the drug target groupings and candidate drugs, but not unrelated drugs, are discordant with coronavirus disease signatures specifically.

The sdf for the 9 drugs that comprise the drug target groupings were downloaded from DrugBank. The sdf files for the 14 candidate drugs were downloaded from PubChem and Chembl. ChemmineR was used to convert the sdf files to binary chemical fingerprints. The Tanimoto coefficients of the chemical fingerprints between the 9 drugs that comprise the drug target groupings and the 14 candidate drugs for all pairs were computed using the ChemmineR library. The Tanimoto coefficient, also known as the Jaccard similarity, represents the established measure for chemical similarity. The data were then visualized as a heat map using the “gplots” package.

### GitHub repository access

R scripts utilized in data processing can be accessed at https://github.com/AliSajid/Covid19.

### Comparing disease and drug target group signatures

Unsupervised clustering analysis was performed on log fold change values of disease and drug target signatures and heat map was generated using *“pheatmap”* package^122^ in R programming language.

### Biological Pathway analysis

To generate biological pathways associated with the drug targets and disease datasets, the gene list was searched in Reactome v70 (https://reactome.org/). For drug target groupings 1–3, the gene signatures (LFC ≥ 0.85 and ≤ −0.85) were searched and pathways with a minimum of 10 entities (genes), p-value < 0.05, were generated. For drug target groupings 4 and 5, the gene signatures (LFC ≥ 0.85 and ≤ −0.85) were searched and pathways with a minimum of 3 entities (genes), p-value < 0.05, were generated, as fewer genes were input into pathway analysis at this threshold. For disease data, the gene signatures (LFC ≥ 0.5 and ≤ − 0.5) were searched and pathways with a minimum of 6 entities (genes), p-value < 0.05, were generated.

Pathways are organized under their top-level hierarchical identifier. Pathways are then identified at multiple sub-levels. To reduce the number of redundant pathways whilst still allowing for meaningful interpretation of biological pathways, some sub-level pathways are contracted.

## Supplementary Material

Supplement

## Figures and Tables

**Table 1.** Drug target groupings. Drug targets with iLINCS signatures that are in use or under investigation for the treatment of COVID-19 were grouped together if they met a least two of the three following criteria: Canonical Mechanism of Action, referenced from the database DrugBank (https://www.drugbank.ca/); Anatomic Therapeutic Chemical classification, referenced from https://www.whocc.no/atc ddd index/: structural similarity as determined by intracluster Tanimoto coefficient. F = Febratininb; R = Ruxolinitib; B = Bariticinib

Drug Cluster	Drug	Canonical Mechanism of Action	Anatomical Therapeutic Chemical *First Level*	Tanimoto Coefficient
1	Chloroquine Hydroxychloroquine	Toll-like receptor antagonists	Antiparasitic Products, Insecticides and Repellants	0.84
2	Lopinavir Ritonavir	Protease inhibitors	Anti-infective for Systemic Use	0.72
3	Fedratinib Ruxolinitib Bariticinib	JAK inhibitors	Antineoplastic and Immunomodulating Agents	F − R: 0.25 F − B: 0.20 B − R: 0.72
4	Azithromycin	Macrolide antibiotic	Anti-Infective for Systemic Use	1
5	Losartan	Angiotensin receptor blocker antagonist	Cardiovascular System	1

**Table 2.** Candidate repurposable drugs for the treatment of COVID-19.

Drug	DrugBank I.D.	MCF7 Concordance	HA1E Concordance	MOA	ATC Drug Class	Indication
**Drugs with reported antiviral activity.**						
Alvocidib	DB03496	0.92	0.96	CDK inhibitor	Unclassified	Antineoplastic
Genistein	DB01645	0.91	0.93	Tyrosine kinase and topoisomerase-II inhibitor	Unclassified	Antineoplastic; nthelmintic
Ivermectin	DB00602	0.90	0.83	Chloride channel agonist	Anthelmintic	Anthelmintic
**Drugs with no reported antiviral activity.**						
***Clinically Relevant Drugs***						
Idebenone	DB09081	0.88	0.81	Electron donor to mitochondrial electron transport chain	Psychoanaleptic	Leber’s Hereditary Optic Neuropathy
Penicillin V	DB01053	0.97	0.80	Binds penicillin binding proteins, inhibits bacterial cell wall synt hesis	Antibacterials for Systemic Use	Antibiotic

Idelalisib	DB09054	0.88	0.84	Phosphoinositid e 3-kinase inhibitor	Antineoplastic Agent
GSK-1059615	DB11962	0.83	0.84	Phosphoinositid e 3-kinase, mTOR inhibitor 46	Unclassified
AT-9283	DB05169	0.89	0.82	kinase inhibitor	Unclassified
***Experimental Drugs***					
GSK-3 Inhibitor IX	--	0.87	0.85	Inhibitor of glycogen synthase kinase-3a/b 47	Unclassified
AC1MJ3VH	--	0.89	0.82	RNA synthesis and topoisomerase inhibitor 48	Unclassified
CCT-10B	--	0.95	0.90	#Kinase binding, protein kinase binding	Unclassified
CHEMBL2136735	--	0.80	0.96	#Oxidoreductas e activity, cadherin binding	Unclassified
Broad-Sai-595	--	0.82	0.92	#NAPH binding, cadherin binding	Unclassified
BRD-K54343811	--	0.98	0.87	#Protein tyrosine kinase binding, phosphotyrosin e residue binding	Unclassified
