# Supplementary material for: Identification of new drug treatments to combat COVID19: A signature-based approach using iLINCS
Source: Res Sq. 2020 Apr 30:rs.3.rs-25643. Preprint. [Version 1] doi: 10.21203/rs.3.rs-25643/v1 (PMC7336712; doi:10.21203/rs.3.rs-25643/v1)
Supplement: Supplement [file Supplementalmaterial.pdf]

Supplementary Information

Identification of new drug treatments to combat COVID19: A signature-based approach using iLINCS

Sinead M O’Donovan, Hunter Eby, Nicholas D Henkel, Justin Creeden, Ali Imami, Sophie Asah, Xiaolu Zhang, Xiaojun Wu, Rawan Alnafisah, R. Travis Taylor, James Reigle, Alexander Thorman, Behrouz Shamsaei, Jarek Meller, Robert E McCullumsmith

Supplemental Figure Legends

Figure S1. Tanimoto coefficient correlogram. Heat map showing the Tanimoto coefficients of the 9 drugs with antiviral indications that comprise drug groupings 1-5 (x-axis) and the 14 candidate repurposable drugs identified in this study (y-axis). The Tanimoto coefficient, which represents the degree of structural similarity between two compounds, was computed using binary chemical fingerprints for each of the drugs. Coefficients range from 0 (no structural similarity) to 1 (exact structural similarity). In this analysis, Tanimoto coefficients ranged from 0.1 - 0.59, suggesting low to moderate structural similarity between antiviral treatments currently in use for treating coronavirus family pathogens and the identified candidate drugs.

Figure S2. Heat map of disease signatures. Unsupervised clustering of Library of Integrated Network-Based Cellular Signature (LINCS) L1000 genes, extracted from RNAseq datasets of severe acute respiratory syndrome (SARS) and Middle East respiratory syndrome-related coronavirus (MERS) (GSE56192). L1000 genes were also extracted from an Influenza A (INFL) microarray dataset (GSE47963) as a non-coronavirus pathogen comparison group. This heat map shows clustering of coronavirus pathogens SARS and MERS in contrast to INFL.

Figure S3. **Heat map of drug groupings 1-5 (MCF7 cell line).** Unsupervised clustering of Library of Integrated Network-Based Cellular Signature (LINCS) L1000 genes, extracted from RNAseq datasets of severe acute respiratory syndrome (SARS) and Middle East respiratory syndrome-related coronavirus (MERS) (GSE56192). L1000 genes were also extracted from an Influenza A (INFL) microarray dataset (GSE47963) as a non-coronavirus pathogen comparison group. L1000 genes were also extracted from five drug groupings (drug clusters 1-5) which represent drugs currently in use to treat coronavirus pathogens. Drug clusters signatures where extracted from the MCF7 cell line. Drugs are clustered based on mechanism of action, ATC

classification and/or structural similarity. Furosemide (FUR), a drug which is not utilized in the treatment of viral pathogens, was utilized as a comparison group. Drug group signatures were calculated by taking the average logFoldChange of each of the drugs within the cluster to represent the gene expression. This heat map provides an overview of the difference in patterns of gene expression between drug target groupings and disease in the MCF7 cell line, demonstrating discordance between drugs used as antivirals and disease signatures. Drug cluster 1: chloroquine and hydroxychloroquine, Drug cluster 2: Lopinavir and Ritonavir, Drug cluster 3: Fedratinib, Ruxolinitib, and Bariticinib. Drug cluster 4: azithromycin, and Drug cluster 5: Losartan.

Figure S4. **Heat map of drug target groupings 1-5 (HA1E cell line).** Unsupervised clustering of Library of Integrated Network-Based Cellular Signature (LINCS) L1000 genes, extracted from RNAseq datasets of severe acute respiratory syndrome (SARS) and Middle East respiratory syndrome-related coronavirus (MERS) (GSE56192). L1000 genes were also extracted from an Influenza A (INFL) microarray dataset (GSE47963) as a non-coronavirus pathogen comparison group. L1000 genes were also extracted from five drug groupings (drug clusters 1-5) which represent drugs currently in use to treat coronavirus pathogens. Drug clusters signatures where extracted from the HA1E cell line. Drugs are clustered based on mechanism of action, ATC classification and/or structural similarity. Furosemide (FUR), a drug which is not utilized in the treatment of viral pathogens, was utilized as a comparison group. Drug group signatures were calculated by taking the average logFoldChange of each of the drugs within the cluster to represent the gene expression. This heat map provides an overview of the difference in patterns of gene expression between drug target groupings and disease in the HA1E cell line, demonstrating discordance between drugs used as antivirals and disease signatures. Drug cluster 1: chloroquine and hydroxychloroquine, Drug cluster 2: Lopinavir and Ritonavir, Drug

cluster 3: Fedratinib, Ruxolinitib, and Bariticinib. Drug cluster 4: azithromycin, and Drug cluster 5: Losartan.

Figure S5. **Biological Pathway analysis of disease and drug target grouping signatures from MCF7 cell line.** Gene lists for drug target groupings 1-5 consisting of L1000 genes at LFC +/- 0.85 were searched in Reactome. Pathways for drug groupings 1 - 3 were determined by p-value < 0.05, and “Entities Found” ≥ 10, and groupings 4 and 5 were determined by p-value < 0.05, and “Entities Found” ≥ 3. Gene lists for disease signatures consisting of L1000 genes at LFC ≥ 0.5 and ≤ -0.5 were searched in Reactome. Pathways were determined by p-value < 0.05, and “Entities Found” ≥ 6. Drug cluster 1: chloroquine and hydroxychloroquine, Drug cluster 2: Lopinavir and Ritonavir, Drug cluster 3: Fedratinib, Ruxolinitib, and Bariticinib. Drug cluster 4: azithromycin, and Drug cluster 5: Losartan.

Figure S6. Biological Pathway analysis of disease and drug target grouping signatures from HA1E cell line. Gene lists for drug target groupings 1-5 consisting of L1000 genes at LFC +/- 0.85 were searched in Reactome. Pathways for drug groupings 1 - 3 were determined by p-value < 0.05, and “Entities Found” ≥ 10, and groupings 4 and 5 were determined by p-value < 0.05, and “Entities Found” ≥ 3. Gene lists for disease signatures consisting of L1000 genes at LFC ≥ 0.5 and ≤ -0.5 were searched in Reactome. Pathways were determined by p-value < 0.05, and “Entities Found” ≥ 6. Drug cluster 1: chloroquine and hydroxychloroquine, Drug cluster 2: Lopinavir and Ritonavir, Drug cluster 3: Fedratinib, Ruxolinitib, and Bariticinib. Drug cluster 4: azithromycin, and Drug cluster 5: Losartan.

Figure S7. **Heat map of 14 candidate drugs (MCF7 cell line).** Unsupervised clustering of Library of Integrated Network-Based Cellular Signature (LINCS) L1000 genes, extracted from RNAseq datasets of severe acute respiratory syndrome (SARS) and Middle East respiratory

syndrome-related coronavirus (MERS) (GSE56192). L1000 genes were also extracted from an Influenza A (INFL) microarray dataset (GSE47963) as a non-coronavirus pathogen comparison group. L1000 gene signatures for the fourteen candidate drugs were generated from MCF7 cell line. Furosemide (FUR), a drug which is not utilized in the treatment of viral pathogens, was utilized as a comparison group. A heat map was constructed using the logFoldChange from LINCS. This heat map provides an overview of the difference in patterns of gene expression between the candidate drugs and disease in the MCF7 cell line, demonstrating discordance between these putative repurposable drugs and coronoavirus disease signatures. AC1MJ3VH (AC1), AT-9283 (AT), Alvocidib (ALV), BRD-K54343811 (BRD), Broad-Sai (BRO), CHEMBL2136735 (CHE), COT-10B (COT), Genistein (GEN), GSK-1059615 (GSK), GSK3 Inhibitor-IX (GIX), Idebenone (IDB), Idelalisib (IDL), Ivermectin (IVE), Pencillin V (PEN).

Figure S8. Heat map of top 8 candidate drugs (MCF7 cell line). Unsupervised clustering of Library of Integrated Network-Based Cellular Signature (LINCS) L1000 genes, extracted from RNAseq datasets of severe acute respiratory syndrome (SARS) and Middle East respiratory syndrome-related coronavirus (MERS) (GSE56192). L1000 genes were also extracted from an Influenza A (INFL) microarray dataset (GSE47963) as a non-coronavirus pathogen comparison group. L1000 gene signatures for the 8 candidate drugs that are currently approved for use in humans were generated from MCF7 cell line. Furosemide (FUR), a drug which is not utilized in the treatment of viral pathogens, was utilized as a comparison group. A heat map was constructed using the logFoldChange from LINCS. This heat map provides an overview of the difference in patterns of gene expression between the candidate drugs and disease in the MCF7 cell line, demonstrating discordance between these putative repurposable drugs and coronoavirus disease signatures. AT-9283 (AT), Alvocidib (ALV), Genistein (GEN), GSK-1059615 (GSK), Idebenone (IDB), Idelalisib (IDL), Ivermectin (IVE), Pencillin V (PEN)

Figure S9. Heat map of 14 candidate drugs (HA1E cell line). Unsupervised clustering of Library of Integrated Network-Based Cellular Signature (LINCS) L1000 genes, extracted from RNAseq datasets of severe acute respiratory syndrome (SARS) and Middle East respiratory syndrome-related coronavirus (MERS) (GSE56192). L1000 genes were also extracted from an Influenza A (INFL) microarray dataset (GSE47963) as a non-coronavirus pathogen comparison group. L1000 gene signatures for the fourteen candidate drugs were generated from HA1E cell line. Furosemide (FUR), a drug which is not utilized in the treatment of viral pathogens, was utilized as a comparison group. A heat map was constructed using the logFoldChange from LINCS. This heat map provides an overview of the difference in patterns of gene expression between the candidate drugs and disease in the HA1E cell line, demonstrating discordance between these putative repurposable drugs and coronoavirus disease signatures. AC1MJ3VH (AC1), AT-9283 (AT), Alvocidib (ALV), BRD-K54343811 (BRD), Broad-Sai (BRO), CHEMBL2136735 (CHE), COT-10B (COT), Genistein (GEN), GSK-1059615 (GSK), GSK3 Inhibitor-IX (GIX), Idebenone (IDB), Idelalisib (IDL), Ivermectin (IVE), Pencillin V (PEN).

Figure S10. Heat map of top 8 candidate drugs (HA1E cell line). Unsupervised clustering of Library of Integrated Network-Based Cellular Signature (LINCS) L1000 genes, extracted from RNAseq datasets of severe acute respiratory syndrome (SARS) and Middle East respiratory syndrome-related coronavirus (MERS) (GSE56192). L1000 genes were also extracted from an Influenza A (INFL) microarray dataset (GSE47963) as a non-coronavirus pathogen comparison group. L1000 gene signatures for the 8 candidate drugs that are currently approved for use in humans were generated from HA1E cell line. Furosemide (FUR), a drug which is not utilized in the treatment of viral pathogens, was utilized as a comparison group. A heat map was constructed using the logFoldChange from LINCS. This heat map provides an overview of the difference in patterns of gene expression between the candidate drugs and disease in the HA1E

cell line, demonstrating discordance between these putative repurposable drugs and coronoavirus disease signatures. AT-9283 (AT), Alvocidib (ALV), Genistein (GEN), GSK-1059615 (GSK), Idebenone (IDB), Idelalisib (IDL), Ivermectin (IVE), Pencillin V (PEN).

Figure S11. Biological Pathway analysis of disease and candidate repurposable drug signatures from MCF7 cell line. Gene lists for candidate drugs consisting of L1000 genes at LFC +/- 0.85 were searched in Reactome. Pathways were determined by p-value < 0.05, and “Entities Found” ≥ 30. Gene lists for disease signatures consisting of L1000 genes at LFC ≥ 0.5 and ≤ -0.5 were searched in Reactome. Pathways were determined by p-value < 0.05, and “Entities Found” ≥ 6. AC1MJ3VH (AC1), AT-9283 (AT), Alvocidib (ALV), BRD-K54343811 (BRD), Broad-Sai (BRO), CHEMBL2136735 (CHE), COT-10B (COT), Genistein (GEN), GSK-1059615 (GSK), GSK3 Inhibitor-IX (GIX), Idebenone (IDB), Idelalisib (IDL), Ivermectin (IVE), Pencillin V (PEN).

Figure S12. Biological Pathway analysis of disease and candidate repurposable drug signatures from HA1E cell line. Gene lists for candidate drugs consisting of L1000 genes at LFC +/- 0.85 were searched in Reactome. Pathways were determined by p-value < 0.05, and “Entities Found” ≥ 30. Gene lists for disease signatures consisting of L1000 genes at LFC ≥ 0.5 and ≤ -0.5 were searched in Reactome. Pathways were determined by p-value < 0.05, and “Entities Found” ≥ 6. AC1MJ3VH (AC1), AT-9283 (AT), Alvocidib (ALV), BRD-K54343811 (BRD), Broad-Sai (BRO), CHEMBL2136735 (CHE), COT-10B (COT), Genistein (GEN), GSK-1059615 (GSK), GSK3 Inhibitor-IX (GIX), Idebenone (IDB), Idelalisib (IDL), Ivermectin (IVE), Pencillin V (PEN).

Figure S1

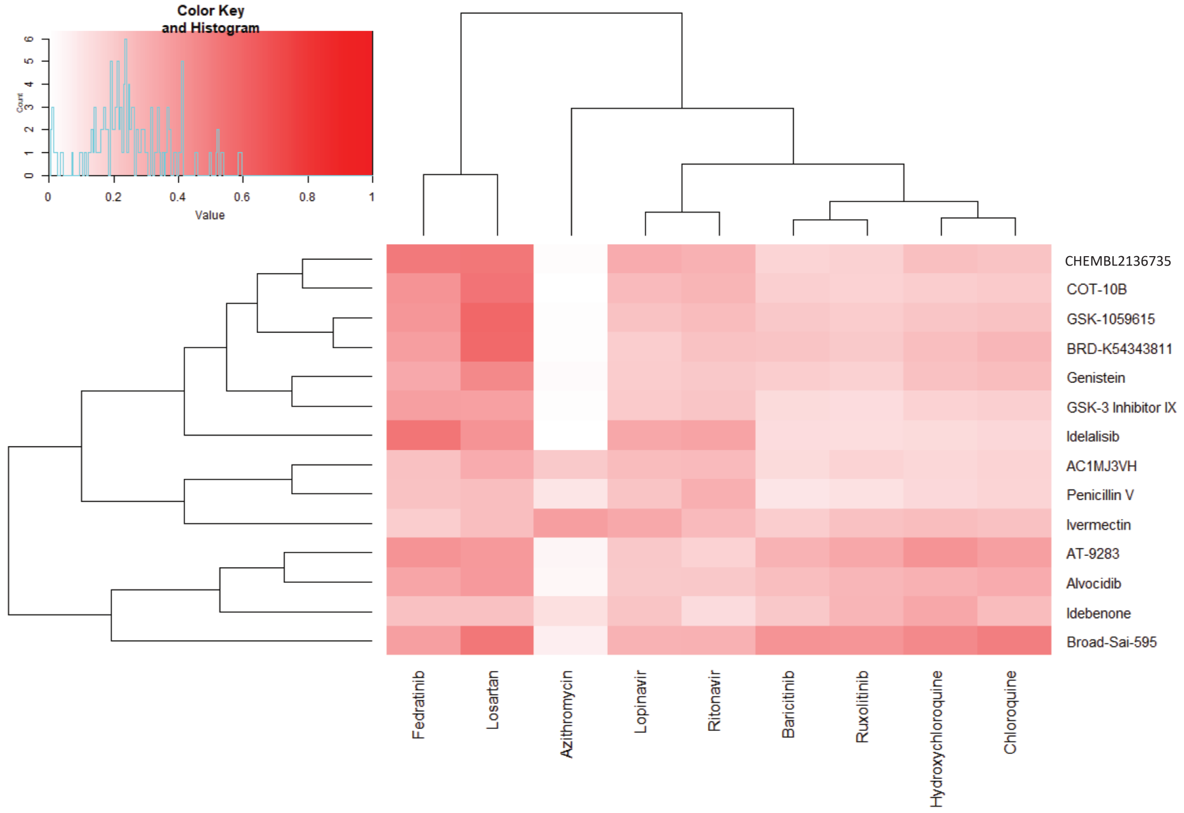
Color Key
and Histogram
Count
0
1
2
3
4
5
6
0
0.2
0.4
0.6
0.8
1
Value
CHEMBL2136735
COT-10B
GSK-1059615
BRD-K54343811
Genistein
GSK-3 Inhibitor IX
Idelalisib
AC1MJ3VH
Penicillin V
Ivermectin
AT-9283
Alvocidib
Idebenone
Broad-Sai-595
Fedratinib
Losartan
Azithromycin
Lopinavir
Ritonavir
Baricitinib
Ruxolitinib
Hydroxychloroquine
Chloroquine

Figure S2

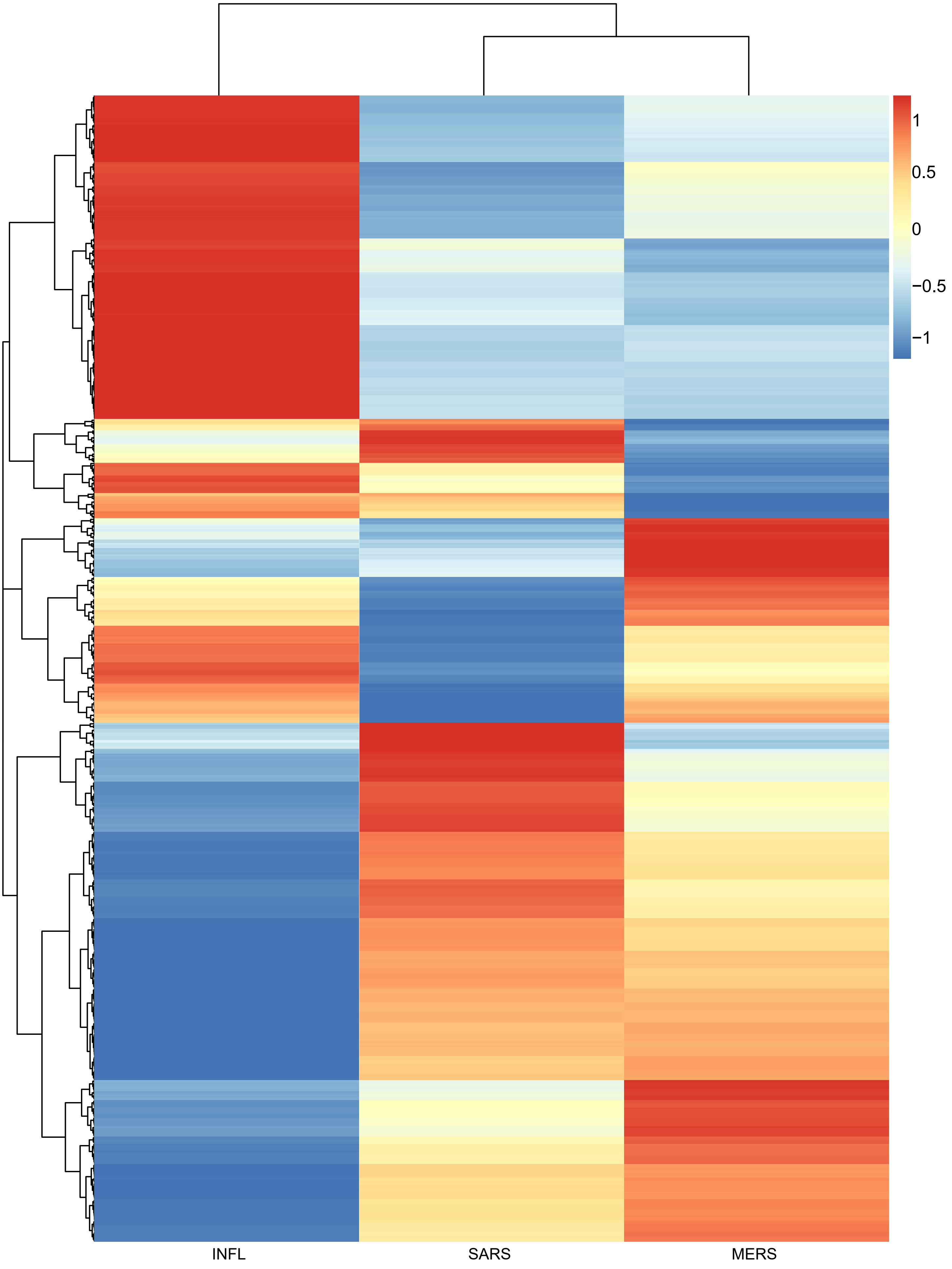

Figure S3

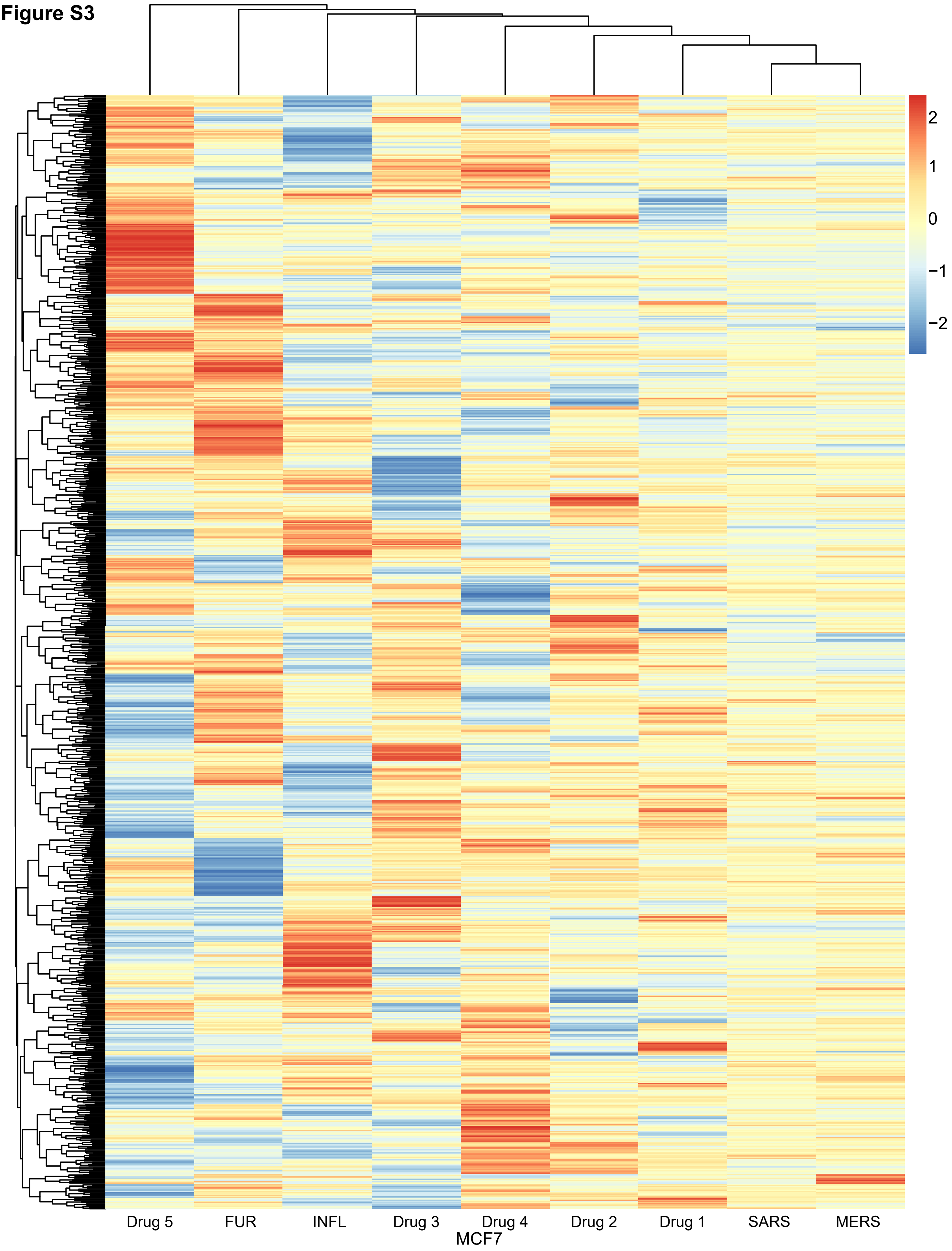

Figure S4

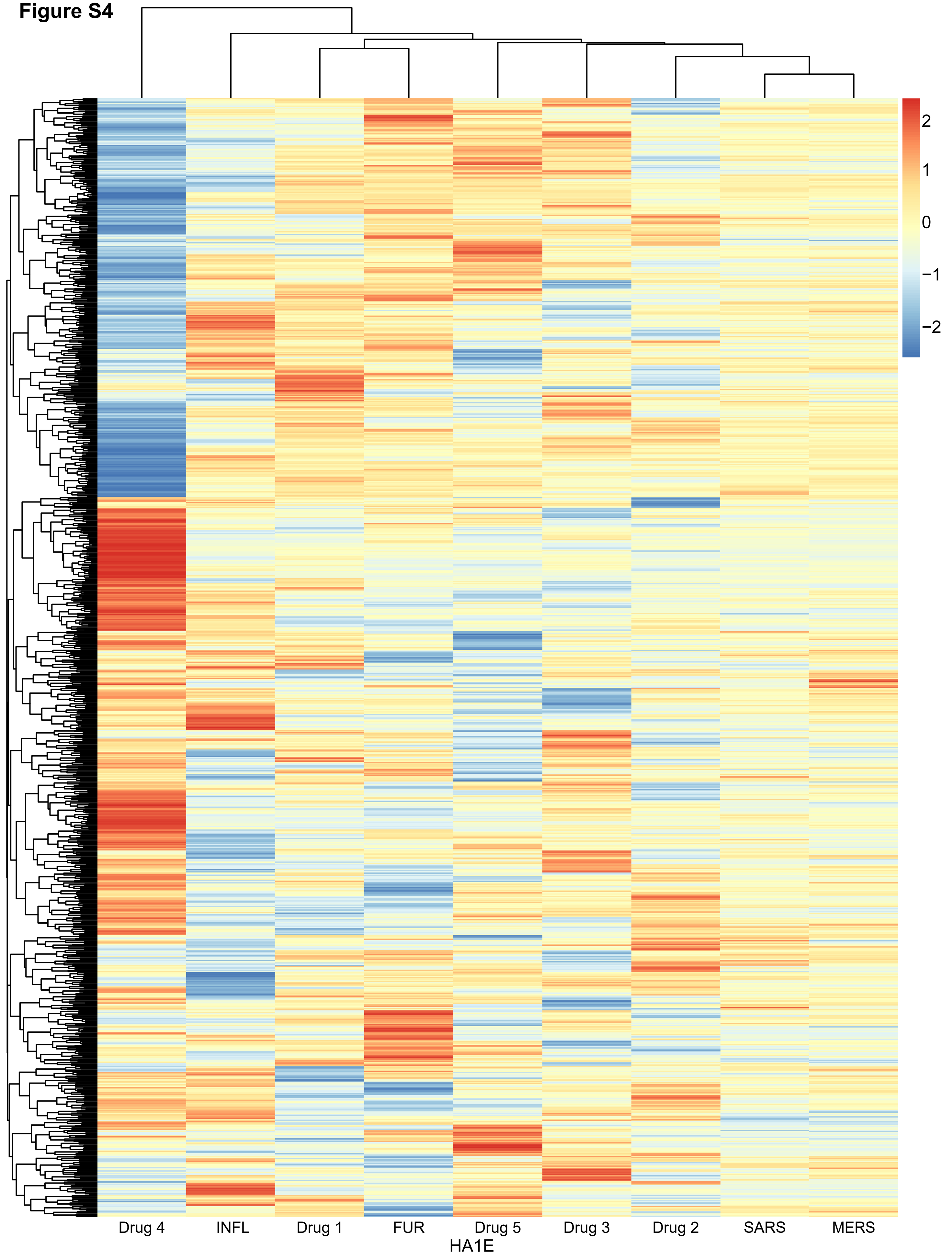

Figure S5

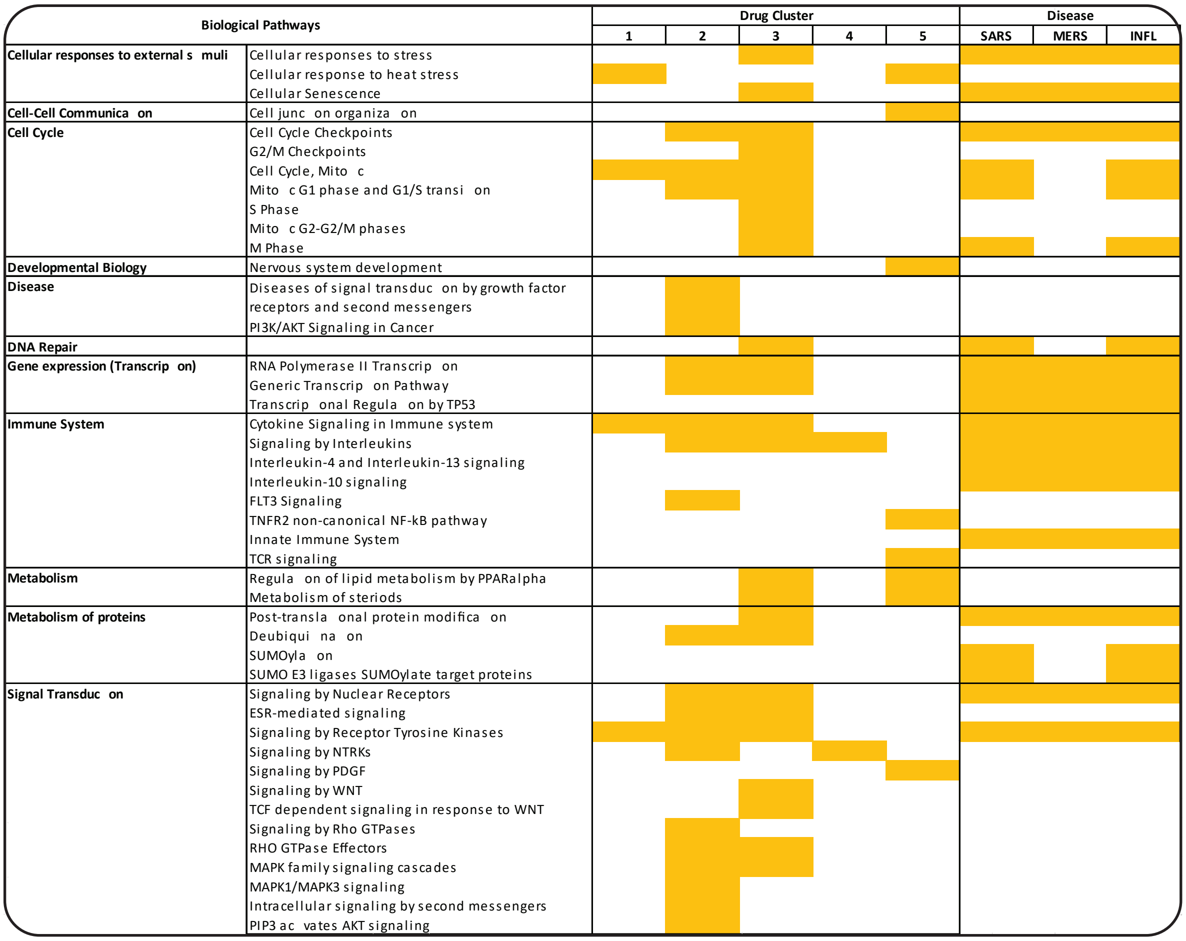

MCF7 Cell Line

Figure S6

Biological Pathways		Drug Cluster					Disease		
		1	2	3	4	5	SARS	MERS	INFL
**Cellular responses to external s muli**	Cellular responses to stress								
	Cellular response to heat stress								
	Cellular Senescence								
**Cell-Cell communica on**									
**Cell Cycle**	Cell Cycle Checkpoints								
	G1/S DNA Damage Checkpoints								
	G2/M Checkpoints								
	Cell Cycle, Mito c								
	Mito c G1 phase and G1/S transi on								
	S Phase								
	Mito c G2-G2/M phases								
	M Phase								
	Regula on of mito c cell cycle								
**Chroma n organiza on**	Chroma n modifying enzymes								
**Developmental Biology**	Nervous system development								
	Axon guidance								
**Disease**	Infec ous disease								
	HIV Infec on								
	Diseases of signal transduc on by growth factor receptors and second messengers								
	PI3K/AKT Signaling in Cancer								
**DNA Repair**	Base Excision Repair								
	Resolu on of Abasic Sites (AP sites)								
	DNA Double-Strand Break Repair								
	Homology Directed Repair								
	Nucleo de Excision Repair								
	Transcrip on-Coupled Nucleo de Excision Repair (TC-NER)								
	Global Genome Nucleo de Excision Repair (GG-NER)								
**DNA Replica on**	Synthesis of DNA								
	DNA strand elonga on								
	Switching of origins to a post-replica ve state								
	DNA Replica on Pre-Ini a on								
**Extracellular matrix organiza on**	Degrada on of the extracellular matrix								
**Gene expression (Transcrip on)**	RNA Polymerase II Transcrip on								
	Generic Transcrip on Pathway								
	Transcrip onal Regula on by TP53								
	Regula on of TP53 Ac vity								
	Epigene c regula on of gene expression								
**Hemostasis**	Platelet ac va on, signaling and aggrega on								
**Immune System**	Cytokine Signaling in Immune system								
	Signaling by Interleukins								
	Interleukin-4 and Interleukin-13 signaling								
	Interleukin-10 signaling								
	FLT3 Signaling								
	Interferon Signaling								
	Innate Immune System								
	Neutrophil degranula on								
	Toll-like Receptor Cascades								
	C-type lec n receptors (CLRs)								
	Fc epsilon receptor (FCERI) signaling								
	Adap ve Immune System								
	Signaling by the B Cell Receptor (BCR)								
**Metabolism**	Metabolism of lipids								
	Regula on of lipid metabolism by PPARalpha								
	Metabolism of carbohydrates								
	Glucose metabolism								
**Metabolism of proteins**	Post-transla onal protein modifica on								
	Deubiqui na on								
	SUMOyla on								
	SUMO E3 ligases SUMOylate target proteins								
	Neddyla on								
	Unfolded Protein Response (UPR)								
	IRE1alpha ac vates chaperones								
**Metabolism of RNA**	Regula on of mRNA stability by proteins that bind AU-rich elements								
**Programmed Cell Death**	Apoptosis								
	Intrinsic Pathway for Apoptosis								
**Signal Transduc on**	Signaling by Nuclear Receptors								
	ESR-mediated signaling								
	Signaling by Receptor Tyrosine Kinases								
	Signaling by NTRKs								
	Signaling by VEGF								
	MAPK family signaling cascades								
	MAPK1/MAPK3 signaling								
	MAPK6/MAPK4 signaling								
	Intracellular signaling by second messengers								
	PIP3 ac vates AKT signaling								
	Signaling by NOTCH								
	Signaling by WNT								
	TCF dependent signaling in response to WNT								
	Signaling by Non-Receptor Tyrosine Kinases								
	Signaling by PTK6								
	Signaling by Hedgehog								
	Hedgehog 'off' state								

HA1E Cell Line

Figure S7

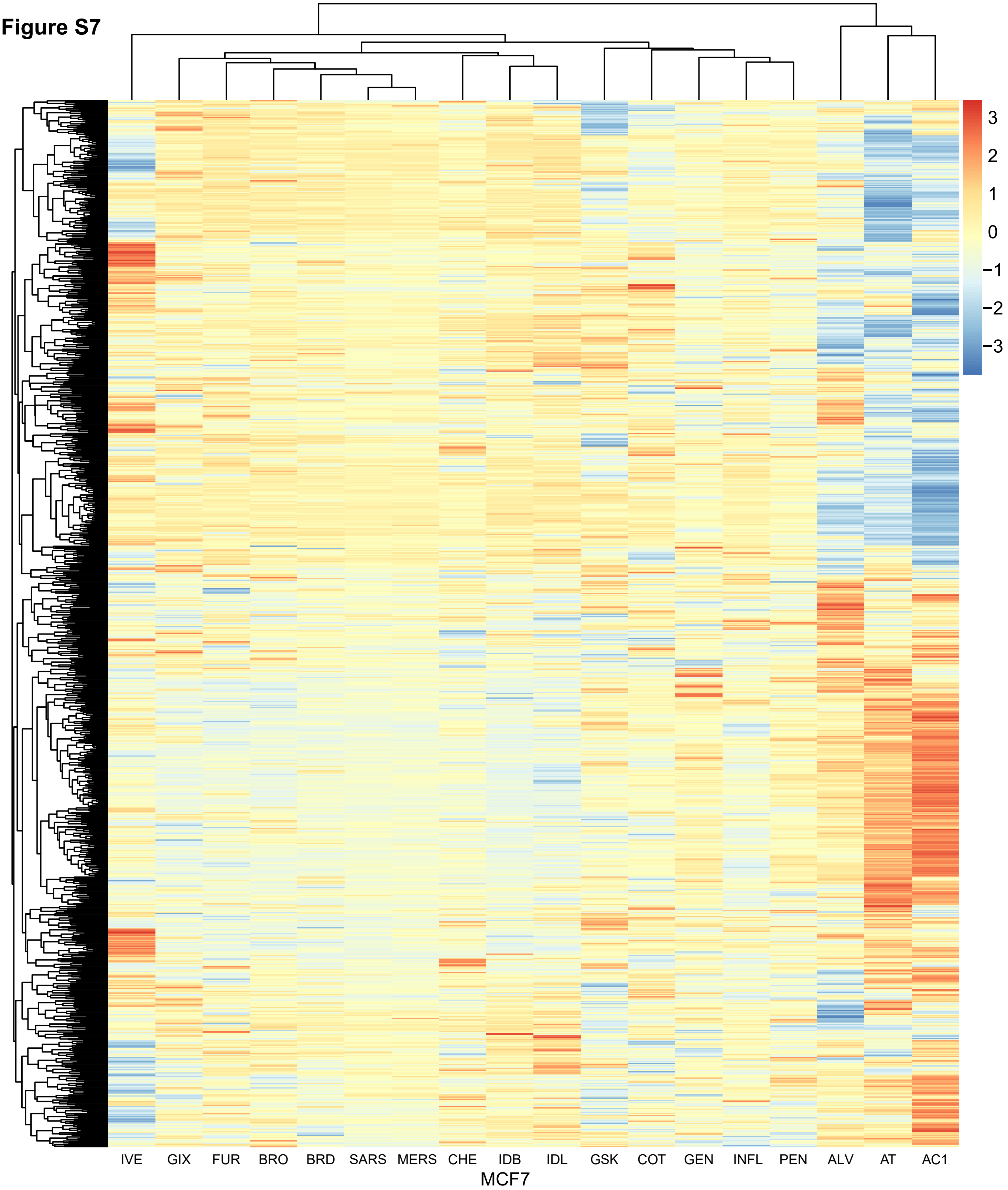
3
2
1
0
−1
−2
−3
IVE
GIX
FUR
BRO
BRD
SARS
MERS
CHE
IDB
IDL
GSK
COT
GEN
INFL
PEN
ALV
AT
AC1
MCF7

Figure S8

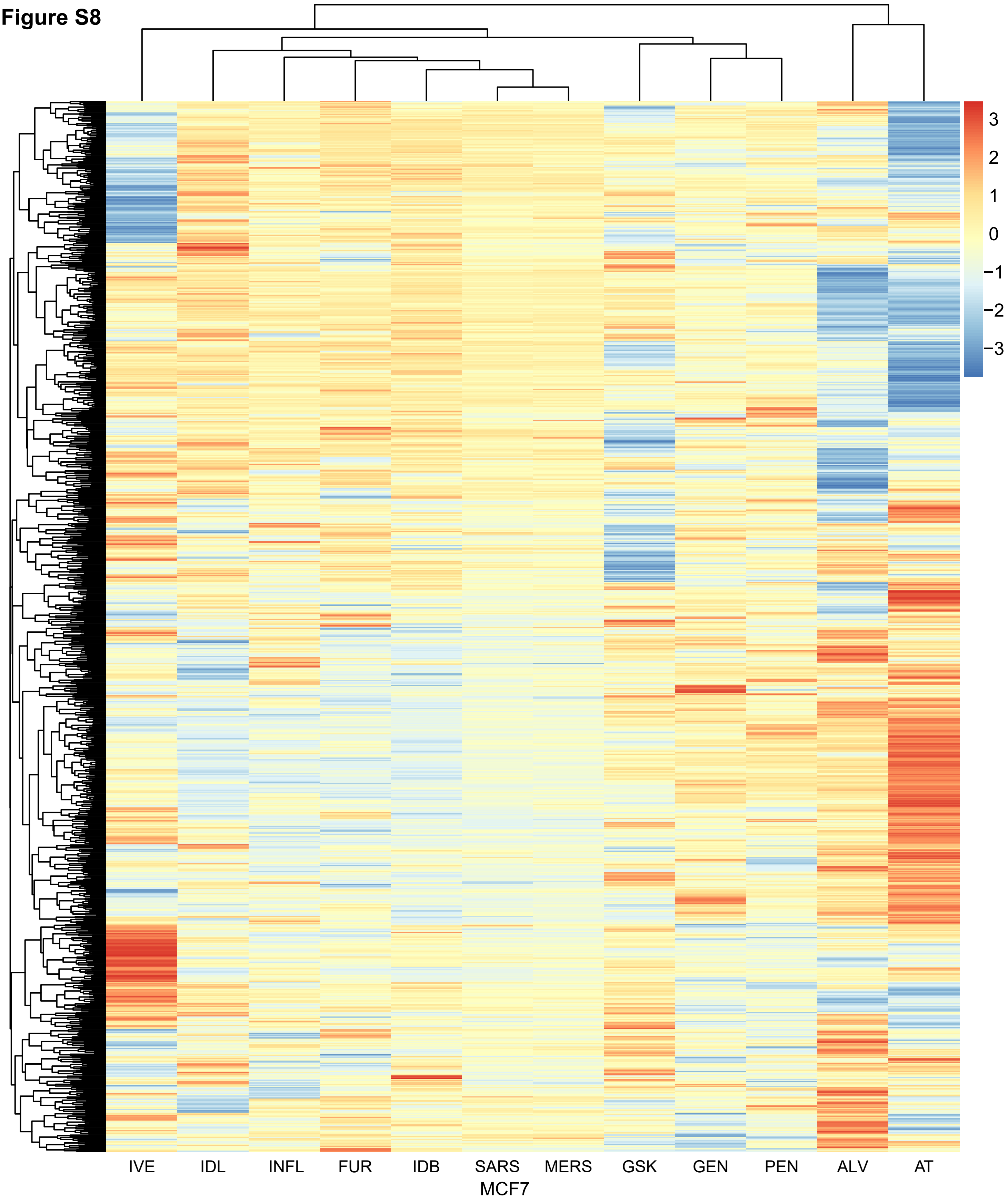

Figure S9

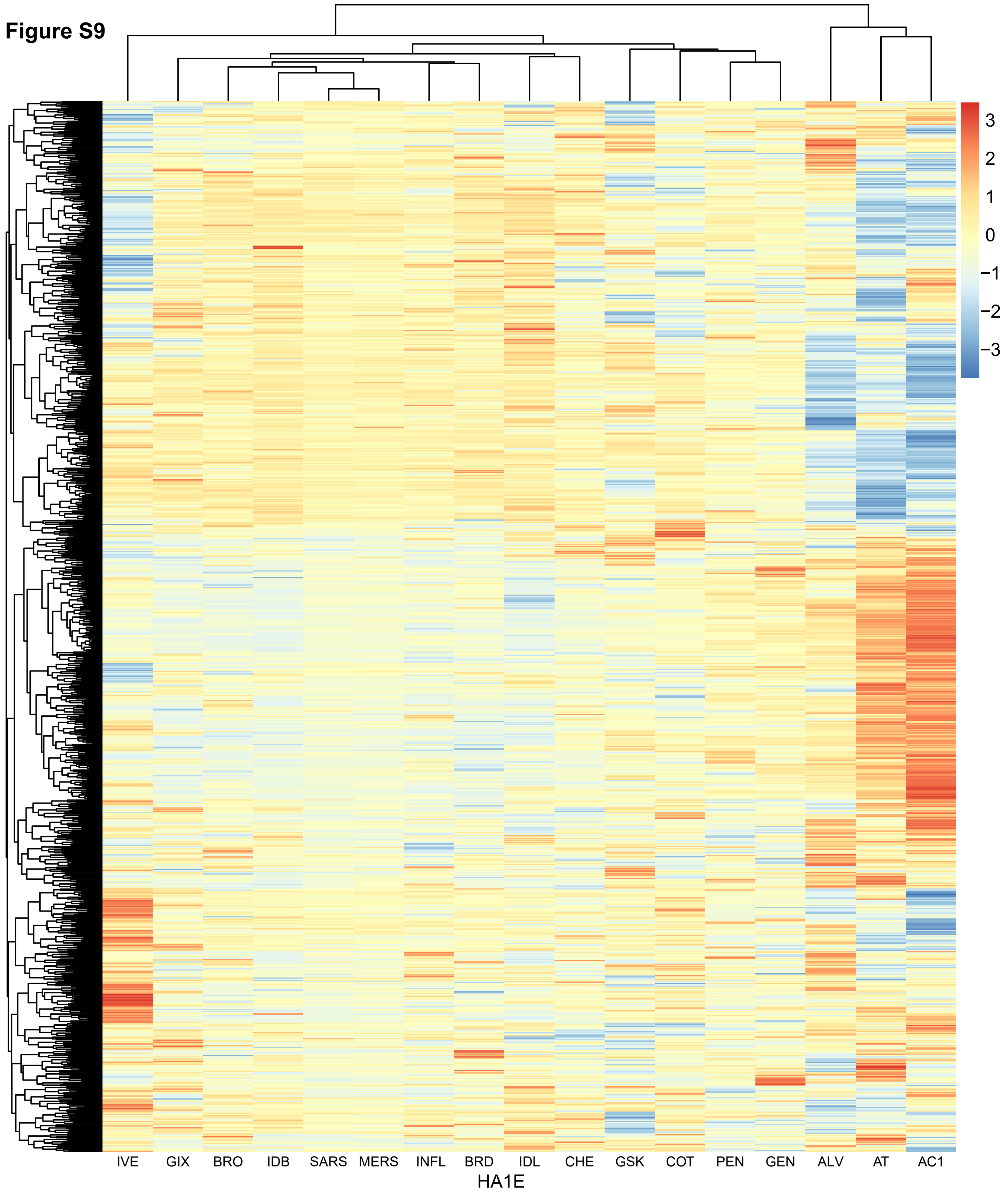
IVE
GIX
BRO
IDB
SARS
MERS
INFL
BRD
IDL
CHE
GSK
COT
PEN
GEN
ALV
AT
AC1
HA1E
3
2
1
0
−1
−2
−3

Figure S10

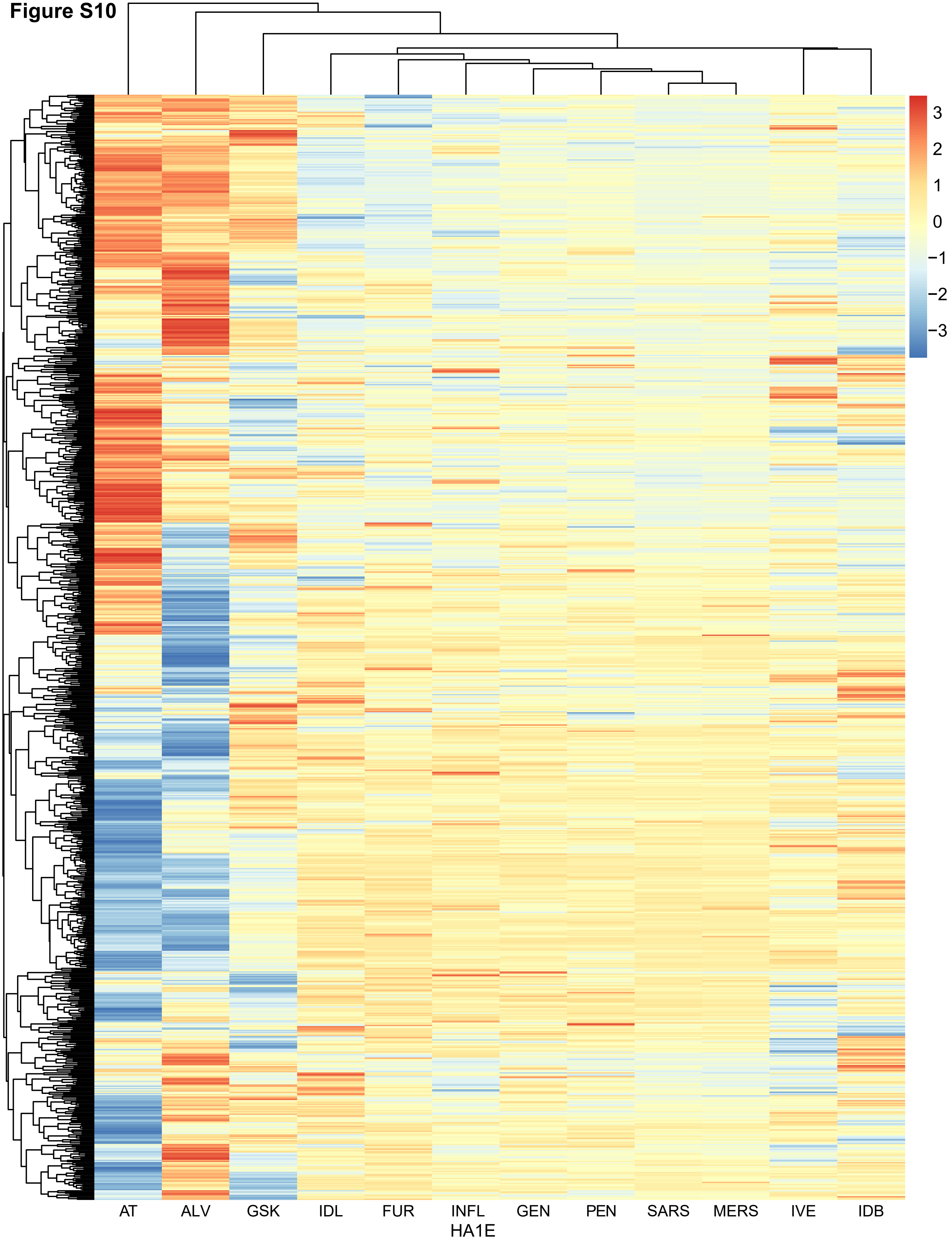

Figure S11

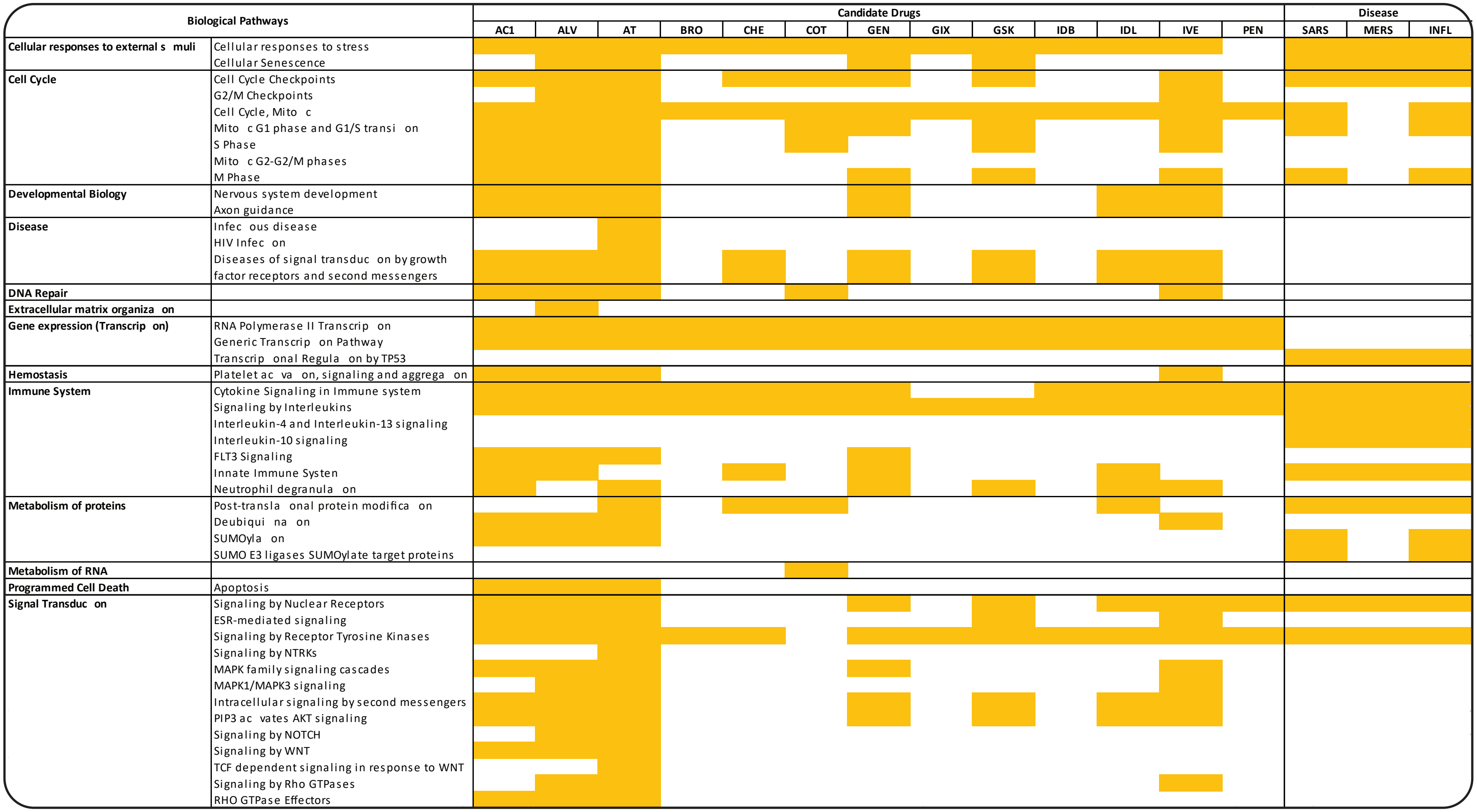

MCF7 Cell Line

Figure S12

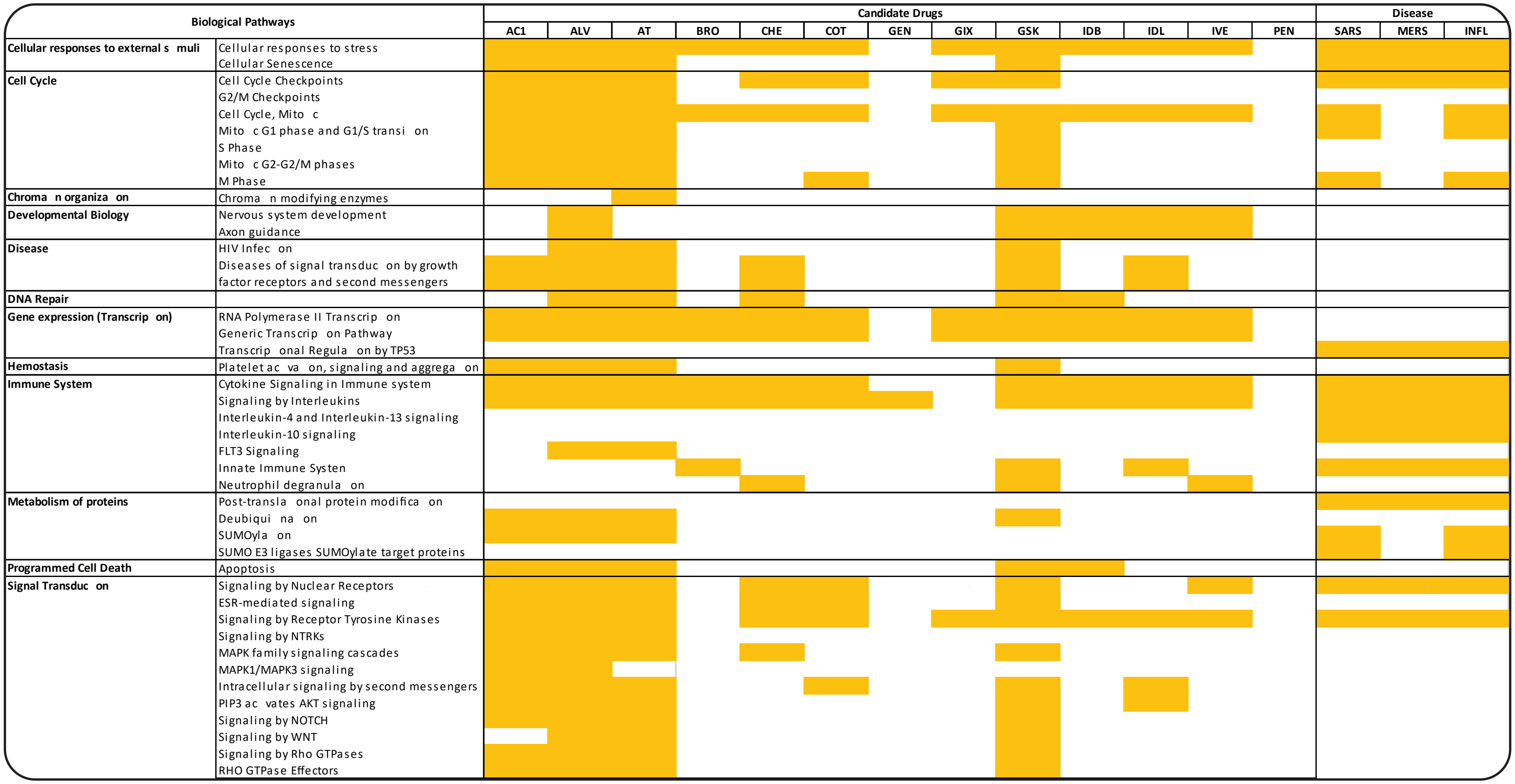

HA1E Cell Line

Table S1. Selected drugs with transcriptional profiles in the Library for Integrated Network-Based Cellular Signatures database which were used for input.

Drug	Trade Names	Clinical Indication	Target	Canonical Mechanism of Action and Clusters	Anatomical Chemical Therapeutic Classification	Literature Citing Relevance to SARS-CoV-2 and/or Coronaviridae	Putative Effect on SARS-CoV-2
Drugs with iLINCS signature present.							
Hydroxychloroquine [1]	Plaquenil	Malaria; Immunosuppressant	Toll-like receptor 9; Toll-like receptor 7	Toll-like receptor antagonist	Antiparasitic products, insecticides and repellents	[2-4]	Alkalization of phagolysosome; modification of post-translation protein modifications [5, 6]
Chloroquine [7]	Aralen	Malaria; Amebiasis	Toll-like receptor 9 Glutathione S-transferase (A2); High mobility group protein B1; tumor necrosis factor				
Lopinavir [8]	Kaletra / Aluvia (with Ritonavir)	HIV/AIDS Antiviral Therapy	Human immunodeficiency virus type 1 protease	Protease inhibitor	Antiinfectives for systemic use	[9-12]	Inhibition of CoV-polyprotein processing [9, 13]
Ritonavir [14]	Norvir; Kaltera /Aluvia (with Lopinavir)						
Fedratinib [15]	Inrebic	Antineoplastic	Tyrosine-protein kinase JAK2; Receptor-type tyrosine-protein kinase FLT3	JAK Inhibitor	Antineoplastic and immunomodulating agents	[16]	Inhibition of clathrin-mediated viral endocytosis; mediation of inflammatory response, attenuation of cytokine storms from prolonged infection [16, 17]
Ruxolitinib [18]	Jakafi, Jakavi	Antineoplastic	Tyrosine-protein kinase JAK1 and JAK2				
Baricitinib [19]	Olumiant, Baricinix	Immunosuppressant	Tyrosine-protein kinase JAK1, JAK2, and JAK3; Protein-tyrosine kinase 2-beta				
Azithromycin [20]	Act Azithromycin, AzaSITE,	Bacterial infections	23S Ribosomal RNA; Protein-arginie deaminase type-4	Inhibition of bacterial protein synthesis	Antiinfectives for systemic use	[4]	Adjunct therapy with hydroxychloroquine.

	Zithromax, Zmax						
Losartan [21]	Act Losartan, Cozaar	Hypertension	Type-1 angiotensin II (AGII) receptor	AGII receptor antagonist	Cardiovascular system	[22]	Angiotensin receptor blocker.
Drugs with iLINCS signature not present.							
Remdesivir [23]	Remdesivir	Ebola	Replicase polyprotein 1ab; RNA-directed RNA polymerase L	Nucleoside analog, inhibition of viral RNA polymerase	No record	[24-27]	Inhibition of RNA dependent RNA polymerase [27]
Rocaglate	Not found in DrugBank.				No record	[28]	Inhibition of viral translation [28].
Silvesterol	Not found in DrugBank.				No record	[28, 29]	Inhibition of viral translation [28].
Umifenovir [30]	Arbidol	Viral prophylaxis; Broad spectrum antiviral	Cytochrome P450 3A4, 2E1, 1A2, 2D6, 2C9, 3A5; UDP-glucuronosyltransferase 2B7, 1-9; Dimethylaniline monooxygenase 1, 3	Aromatic rings interfere with viral endocytosis, exocytosis, intracellular trafficking, and destabilize membranes	Antiinfectives for systemic use	[31-33]	No specific purported mechanism yet published.
N10169	Not found in DrugBank.				No record	[34]	Inhibition of pyrimidine biosynthesis [34].
Interferon Alphacon-1 [35]	Infergen	Antineoplastic	Interferon alpha/beta receptor 1, 2	Interferon receptor agonist, JAK activator	Antineoplastic and immunomodulating agents	[36, 37]	Upregulation of MHC 1 to present viral antigens.
Polygonaceae spp.; Rheum palmatum L.	Not found in DrugBank.				No record	[38, 39]	Inhibition of viral cell-entry by preventing interaction of CoV Spike Protein from interacting with ACE2 receptor [39]
Camostat mesylate [40]	N/A	Pancreatitis, Japan [41, 42]	N/A	Serine protease inhibitor TMPRSS2	Blood and blood forming organs	[43-45]	Inhibition of viral cell-entry [43]

Information relating to these drugs (trade name, clinical indication, targets, and canonical mechanism of action) were referenced from DrugBank (https://www.drugbank.ca/). Anatomical Therapeutic Chemical (ATC) classification was referenced from (https://www.whocc.no/atc_ddd_index/). For simplicity, the first-level of ATC classification is indicated.

Table S2. Gene threshold cutoff of generating disease signatures**.**

Disease	Cell Line	Perturbagens Identified at LFC threshold		
		All L1000	0.26	0.5
**INFL**	HA1E	1	0	0
	MCF7	0	0	0
**MERS**	HA1E	2	28	320
	MCF7	14	142	1305
**SARS**	HA1E	20	172	1352
	MCF7	21	706	2181

L1000 genes with expression change at log fold change (LFC) threshold 0.5 were selected to generate severe acute respiratory syndrome (SARS), Middle East respiratory syndrome (MERS), and influenza (INFL) gene signatures. At this threshold, following connectivity analysis, a large number of chemical perturbagen signatures were identified as discordant to the SARS signature (discordance $\leq$ -0.321) in both MCF7 and HA1E cell lines. At the same threshold, no chemical perturbagens were identified as discordant to the INFL signature, and fewer than 50% of the number of perturbagens were identified in MERS compared to SARS.
